# Coffee consumption and cardiometabolic health: a comprehensive review of the evidence

**DOI:** 10.1007/s11357-024-01262-5

**Published:** 2024-07-04

**Authors:** Zoltan Ungvari, Setor K. Kunutsor

**Affiliations:** 1 Vascular Cognitive Impairment, Neurodegeneration and Healthy Brain Aging Program, Department of Neurosurgery, University of Oklahoma Health Sciences Center, Oklahoma City, OK USA; 2 Stephenson Cancer Center, University of Oklahoma, Oklahoma City, OK USA; 3 Oklahoma Center for Geroscience and Healthy Brain Aging, University of Oklahoma Health Sciences Center, Oklahoma City, OK USA; 4 Department of Health Promotion Sciences, College of Public Health, University of Oklahoma Health Sciences Center, Oklahoma City, OK USA; 5 International Training Program in Geroscience, Doctoral College/Department of Preventive Medicine and Public Health, Semmelweis University, Budapest, Hungary; 6 Leicester Real World Evidence Unit, Diabetes Research Centre, University of Leicester, Leicester General Hospital, Gwendolen Road, Leicester, LE5 4WP UK; 7 Section of Cardiology, Department of Internal Medicine, Rady Faculty of Health Sciences, University of Manitoba, Saint Boniface Hospital, Winnipeg, MB R2H 2A6 Canada

**Keywords:** Coffee consumption, Caffeine, Cardiometabolic, Type 2 diabetes, Hypertension, Chronic kidney disease, Cardiovascular disease, Mortality

## Abstract

This review provides a comprehensive synthesis of longitudinal observational and interventional studies on the cardiometabolic effects of coffee consumption. It explores biological mechanisms, and clinical and policy implications, and highlights gaps in the evidence while suggesting future research directions. It also reviews evidence on the causal relationships between coffee consumption and cardiometabolic outcomes from Mendelian randomization (MR) studies. Findings indicate that while coffee may cause short-term increases in blood pressure, it does not contribute to long-term hypertension risk. There is limited evidence indicating that coffee intake might reduce the risk of metabolic syndrome and non-alcoholic fatty liver disease. Furthermore, coffee consumption is consistently linked with reduced risks of type 2 diabetes (T2D) and chronic kidney disease (CKD), showing dose-response relationships. The relationship between coffee and cardiovascular disease is complex, showing potential stroke prevention benefits but ambiguous effects on coronary heart disease. Moderate coffee consumption, typically ranging from 1 to 5 cups per day, is linked to a reduced risk of heart failure, while its impact on atrial fibrillation remains inconclusive. Furthermore, coffee consumption is associated with a lower risk of all-cause mortality, following a U-shaped pattern, with the largest risk reduction observed at moderate consumption levels. Except for T2D and CKD, MR studies do not robustly support a causal link between coffee consumption and adverse cardiometabolic outcomes. The potential beneficial effects of coffee on cardiometabolic health are consistent across age, sex, geographical regions, and coffee subtypes and are multi-dimensional, involving antioxidative, anti-inflammatory, lipid-modulating, insulin-sensitizing, and thermogenic effects. Based on its beneficial effects on cardiometabolic health and fundamental biological processes involved in aging, moderate coffee consumption has the potential to contribute to extending the healthspan and increasing longevity. The findings underscore the need for future research to understand the underlying mechanisms and refine health recommendations regarding coffee consumption.

## Introduction

Adverse cardiometabolic outcomes, encompassing hypertension, metabolic syndrome (MetS), nonalcoholic fatty liver disease (NAFLD), type 2 diabetes (T2D), chronic kidney disease (CKD), and cardiovascular diseases (CVDs), represent a significant public health burden globally. These diseases significantly impact morbidity, life expectancy, quality of life, and mortality. Hypertension, a leading risk factor for CVD and stroke, affects an estimated 1.28 billion adults globally, with a higher prevalence in low- and middle-income countries [1, 2]. Metabolic syndrome, a complex of interconnected metabolic risk factors that include abdominal obesity, insulin resistance, high blood pressure, and atherogenic dyslipidemia (consists of an aggregation of lipoprotein abnormalities including elevated serum triglyceride and apolipoprotein B (apoB), increased small low-density lipoprotein (LDL) particles, and a reduced level of high-density lipoprotein cholesterol (HDL-C)) [3, 4], is an important contributor to T2D, CVD, and premature death [5–8]. Nonalcoholic fatty liver disease is a cardiometabolic condition which is characterized by hepatic steatosis with varying degrees of necroinflammation and fibrosis [9]. It is a major cause of cirrhosis and hepatocellular carcinoma [9]. Type 2 diabetes, characterized by elevated blood sugar levels, is a common condition contributing to various health complications, including kidney failure, heart disease, and stroke [10]. Chronic kidney disease reveals a troubling burden, in terms of both morbidity and mortality, as well as substantial economic costs associated with its diagnosis and management [11, 12]. Cardiovascular disease, with its major manifestations being coronary heart disease (CHD) and stroke, is the leading cause of death globally [13]. Together, these diseases pose a substantial burden on healthcare systems and global economies. The rising prevalence of these conditions is linked to aging populations[14] and increased exposure to modifiable lifestyle risk factors such as tobacco use, physical inactivity, unhealthy diet, and harmful alcohol consumption [15–17]. These lifestyle choices play a pivotal role in the development and progression of these diseases, highlighting the importance of preventive strategies in public health [18].

The role of dietary factors in mitigating the risk of these cardiometabolic diseases is increasingly recognized. A balanced diet, rich in fruits, vegetables, whole grains, and lean proteins, has been shown to have protective effects against these conditions [19–22]. Amidst various dietary components, coffee consumption emerges as a topic of growing interest due to its widespread use and potential health implications. Coffee, a beverage with a rich history and cultural significance and the most popular and widely consumed beverage in the world [23], has been a subject of numerous studies examining its impact on health [24].

The history of coffee is as rich and robust as the beverage itself, spanning centuries and cultures, with its roots deeply embedded in both social and medicinal contexts [25, 26]. Coffee is believed to have originated in Ethiopia around the ninth century, where its beans were initially chewed for energy by local tribes. The use of coffee as a drink spread to the Arabian Peninsula, and by the sixteenth century, it was known in Persia, Egypt, Syria, and Turkey. Historically, coffee was not only consumed for pleasure but also valued for its medicinal properties [26]. In Arabian culture, coffee was prescribed as a medicine for a variety of ailments from simple headaches to more complex conditions like depression [26]. By the seventeeth century, coffee had made its way to Europe and was sold by apothecaries as a remedy for digestive disorders, a practice that was particularly common in Germany and France. In the 1600s, detailed medicinal reports began appearing on coffee’s beneficial effects, such as its ability to cure certain diseases, aid in digestion, excite mental prowess, and act as a stimulant [26].

The journey of coffee through medical scrutiny has a colorful past [26], highlighted by what might be considered one of the earliest instances of a controlled clinical trial. This experimental approach to understanding coffee’s health effects dates back to the eighteeth century under the rule of Gustav III of Sweden (1746–1792 AD), who had a complex view on the beverage’s safety [27]. The King’s skepticism towards coffee was inherited from a backdrop of stringent regulations instigated by his father, Adolph Frederick, who enacted the “Misuse and Excesses Tea and Coffee Drinking Edict” [28]. This law not only imposed heavy taxes on coffee but also penalized its consumption. In a bold move to investigate health implications of coffee consumption, Gustav III initiated an experiment involving two identical twins convicted of a crime. Their death sentences were commuted to life imprisonment on the condition that they participate in his study, with one twin consuming three pots of coffee daily and the other the same amount of tea. The results were clear when the coffee-drinking twin outlived his tea-consuming counterpart, dying at a later age [27]. This outcome eventually contributed to the lifting of the coffee ban in Sweden during the 1820s.

In modern times, the majority of health practitioners have recommended avoiding coffee in patients with CVD [29], due to side effects such as increased blood pressure (BP) and cardiac arrhythmias [30, 31], that may adversely impact cardiovascular outcomes. In fact, in the 1960s, coffee consumption was proposed as a cardiovascular risk factor [32]. However, recent evidence suggests that coffee consumption may exert beneficial effects on several cardiometabolic outcomes [33, 34]. Studies have shown associations between coffee intake and reduced risk of outcomes such as T2D, CVD, and mortality.[33, 35, 36] However, the evidence has not always been consistent, with some studies suggesting neutral or even adverse effects [33, 37, 38].

Given the extensive literature and several inconsistencies in findings, there is a pressing need to summarize and appraise the evidence surrounding coffee consumption and cardiometabolic health in one single investigation. This will enable patients, clinicians, researchers, and policy makers to make the appropriate interpretations, which can optimally impact on public health and clinical practice. This review aims to provide a comprehensive overview of the current state of evidence, delve into the biological mechanisms through which coffee may exert its cardiometabolic health effects, and discuss the health, clinical, and policy implications. The following adverse cardiometabolic outcomes are evaluated: hypertension, MetS, NAFLD, T2D, CKD, composite CVD, and specific endpoints such as CHD, stroke, heart failure (HF), and atrial fibrillation (AF), and all-cause mortality. This review also discusses the potential of coffee consumption to contribute to the extension of healthspan and improve longevity, based on its benefits to cardiometabolic health. The review also highlights gaps in the existing evidence and suggests future research directions in this area. Additionally, this study reviews evidence on the causal relationships between coffee consumption and these cardiometabolic outcomes using Mendelian randomization (MR) studies. Such a synthesis is very relevant, considering the substantial public health burden attributed to adverse cardiometabolic outcomes and the widespread consumption of coffee globally.

## Methods

A search of MEDLINE and Embase was conducted up to May 2024 for randomized controlled trials (RCTs), non-RCTs, and observational studies, including prospective cohort, nested case-control, case-cohort, or retrospective cohort studies, with a particular focus on systematic reviews and meta-analyses of these study designs, based on the hierarchy of evidence [39]. Search terms or keywords related to coffee consumption (“coffee,” “coffee consumption”) and cardiometabolic outcomes (“hypertension,” “metabolic syndrome,” “NAFLD,” type 2 diabetes,” “chronic kidney disease,” “cardiovascular disease,” “coronary heart disease,” “stroke,” “heart failure,” “atrial fibrillation,” “mortality”) were combined. The review was restricted to studies conducted in human population, reported in English, and in adults. For observational studies, the focus was particularly on longitudinal cohort studies given that they address the issue of temporality. Studies that studied the effect of the combination of coffee and tea/cocoa-based beverages were not evaluated. In a separate search, MR studies on coffee consumption and cardiometabolic outcomes were identified.

## Types of coffee

Coffee is a complex beverage composed of over 100 biological and chemical components, including carbohydrates, lipids, nitrogenous compounds, vitamins, minerals, and a variety of bioactive compounds such as diterpenes, magnesium, trigonelline, quinides, lignans, alkaloids, and phenolic compounds [40]. The principal active ingredient in coffee, caffeine, is the most widely consumed psychostimulant in the world [41]. The composition of these components can vary significantly depending on the coffee bean variety, roasting degree, and brewing method.

There are two primary types of coffee beans: Arabica and Robusta. Arabica beans, which constitute about 70% of the world’s coffee production, are prized for their smooth flavor and aromatic qualities. Robusta beans, making up the remaining 30%, are more robust and bitter, often used in blends for added body and crema. They contain higher levels of caffeine compared to Arabica beans [42].

Coffee can be classified into two subtypes: instant coffee and ground coffee, which differ in preparation, taste, and caffeine content [42]. Instant coffee is created from brewed coffee that has been freeze-dried or spray-dried into soluble powder or granules. To prepare, you simply dissolve it in hot water, making it a quick and convenient option. Instant coffee typically contains between 60 and 80 mg of caffeine per 8-oz cup. It is often made from lower-grade coffee beans. Ground coffee is made from coffee beans that have been roasted and then ground. It is used in various brewing methods, such as drip brewing, French press, or espresso machines. The caffeine content in ground coffee can vary widely, depending on the bean type, roast level, and brewing method. Generally, an 8-oz cup of ground coffee can contain anywhere between 70 and 140mg of caffeine.

Globally, coffee is enjoyed in numerous forms, ranging from traditional brews like espresso, Americano, and French press to more contemporary styles such as latte, cappuccino, macchiato, mocha, flat white, iced coffee, and cold brew [43]. Each preparation method influences the composition as well as the flavor and texture of the final product. Espresso coffee traces its origins back to Turin in 1884, with the invention of the machine known as “La Brasiliana,” patented by Angelo Moriondo (patent No. 33/256 dated May 16, 1884, and later patent No. 34/381 dated November 20, 1884). This innovation was internationally patented in Paris on October 23, 1885 [44]. The term “espresso coffee” first emerged at the 1906 Milan Fair, coined by Desiderio Pavoni to describe this new coffee preparation method [45]. In 1936, Antonio Cremonese officially included “espresso coffee” in a patent (patent No. 343230). This patent was subsequently purchased and enhanced by Achille Gaggia, who marketed the machine as a “crema coffee” machine. The name “crema coffee” referred to the distinctive layer of crema that differentiated it from instant coffees. Thus, crema coffee evolved into the espresso coffee we recognize today [46]. In 1938, Gaggia filed patent No. 365726, which marked a significant advancement in coffee extraction technology. His machine employed a piston system to push high-temperature water through the coffee powder, creating the first pressurized espresso extraction method. This innovation resulted in espresso coffee that was free from traditional bitterness and burnt aftertaste, characterized instead by a thick, creamy texture [45]. In 1947, Gaggia registered a second patent, introducing a lever system that replaced the press mechanism. This lever pushed water at a pressure of 9/10 atmospheres into the ground coffee, allowing for the extraction of aromatic compounds and the formation of crema. The result was a coffee that retained its full olfactory and taste characteristics. The intense aroma and rich flavor profile contributed to the rapid popularity of “crema espresso,” solidifying it as a celebrated symbol of Italian coffee culture [44].

Coffee can also be categorized based on its caffeine content into caffeinated and decaffeinated varieties. The caffeine extraction involves various methods that reduce caffeine levels while attempting to maintain the original flavor profile.

Coffee can broadly be classified into three preparation styles based on how it is brewed. Boiled coffee is one of the oldest methods, where ground coffee is boiled in water, typically in a pot or kettle. This method does not use a filter, allowing the grounds to naturally settle at the bottom.

Unfiltered coffee is a brewing method in which coffee grounds are steeped in hot water and then separated from the liquid using a method that allows some fine particles to remain in the final brew. This method encompasses styles such as Turkish coffee and French press. In Turkish coffee, the finely ground coffee is simmered in a pot with water and often sugar, then served into cups where the grounds are allowed to settle. In the French press, coarser grounds are steeped in hot water, and then a plunger is used to press the grounds to the bottom of the pot, allowing the brewed coffee to remain above the mesh filter.

The filtered coffee method involves brewing by pouring hot water over coffee grounds contained within a filter. As the water percolates through the grounds, it extracts flavors and compounds, but leaves behind most of the coffee particles and oils, thanks to the filter. This process produces a coffee that is lighter in body and cleaner in taste compared to unfiltered coffee methods. One significant characteristic of filtered coffee is that it lacks the rich diterpene compounds found in unfiltered coffee, such as cafestol and kahweol, which are known to contribute to the oiliness and robust flavor of coffees like those made from a French press or Turkish brewing method. The absence of these diterpenes makes filtered coffee a healthier choice for those concerned about cholesterol, as diterpenes have been shown to elevate LDL cholesterol levels [47].

## Coffee consumption and impact on adverse cardiometabolic outcomes

### Blood pressure and hypertension

The relationship between coffee consumption and blood pressure (BP)/hypertension is complex. Coffee consumption has been linked to increases in BP or risk of hypertension, whereas some studies suggest a protective effect of coffee intake. A large number of RCTs and observational cohort studies of the effect of coffee or caffeine consumption on BP or hypertension have been conducted, and there have been several efforts to aggregate the evidence using systematic reviews and meta-analyses. Jee and colleagues [48] in their 1999 meta-analysis of 11 RCTs showed that coffee consumption (median dose of 5 cups/day) was associated with increases in systolic and diastolic blood pressure (SBP and DBP, respectively) (2.4 and 1.2 mmHg, respectively) following a median duration of 56 days. The effect of coffee consumption on SBP and DBP was greater in trials with younger participants [48]. In a meta-analysis of 16 caffeine and coffee consumption RCTs of 42 days median duration published by Noordzij and colleagues [49] in 2005, SBP and DBP were shown to increase by 2.04 and 0.73 mmHg, respectively. When coffee and caffeine trials were analyzed separately, BP elevations appeared to be larger for caffeine (SBP 4.16 mmHg (2.13–6.20) and DBP 2.41 mmHg (0.98–3.84)) than for coffee consumption (SBP 1.22 mmHg (0.52–1.92) and DBP 0.49 mmHg (−0.06–1.04)) [49]. In a 2021 meta-analysis of RCTs to evaluate the effects of coffee consumption on MeTS parameters, Ramli and colleagues [50] showed that green coffee extract supplementation reduced SBP and DBP. Mesas and colleagues [51] in 2011 conducted a meta-analysis of five RCTs to summarize the evidence on the acute and longer-term effects of caffeine and coffee intake on BP in hypertensive individuals. Results showed that the administration of 200–300 mg caffeine produced a mean increase of 8.1 mmHg in SBP and of 5.7 mmHg in DBP. The increase in BP was observed in the first hour after caffeine intake and lasted ≥3 h [51]. In three studies of the longer-term effect (2 weeks) of coffee, no increase in BP was observed after coffee was compared with a caffeine-free diet or was compared with decaffeinated coffee [51].

In a 2017 dose-response meta-analysis of seven observational cohort studies by Grosso and colleagues [52], the nonlinear analysis showed a 9% significant decreased risk of hypertension per 7 cups of coffee a day, while, in the linear dose–response analysis, there was a 1% decreased risk of hypertension for each additional cup of coffee per day. In stratified analysis, significant inverse associations were observed in females, but not in males; however, these analyses need to be interpreted with caution given the limited number of studies for the stratified analysis [52]. In a 2018 dose-response meta-analysis of ten observational cohort studies, Xie and colleagues [53] showed that coffee consumption was weakly and inversely associated with the risk of hypertension in a linear dose-response manner. For the dose-response curve, the relative risks (RRs) of hypertension risk were 0.97 (95% CI, 0.95–0.99), 0.95 (95% CI, 0.91–0.99), 0.92 (95% CI, 0.87–0.98), and 0.90 (95% CI, 0.83–0.97) for 2, 4, 6, and 8 cups/day, respectively, compared with individuals with no coffee intakes [53]. The associations did not vary significantly by age and sex in stratified analyses [53]. In a 2019 meta-analysis by D’Elia and colleagues [54] involving four prospective cohort studies, a nonlinear inverse dose-response relationship was demonstrated between coffee consumption and the risk of hypertension. Compared with no coffee consumption, the RRs of hypertension were 1.00 (95% CI, 0.99–1.01) for 1 cup/day, 0.99 (95% CI, 0.97–1.02) for 2 cups/day, 0.97 (95% CI, 0.94–0.99) for 3–4 cups/day, 0.94 (95% CI, 0.91–0.97) for >4–5 cups/day, 0.90 (95% CI, 0.86–0.93) for >5–6 cups/day, and 0.86 (95% CI, 0.82–0.91) for >6–7 cups/day compared with no coffee consumption [54]. The associations did not vary by age categories [54]. In a 2023 meta-analysis of 12 observational cohort studies by Haghighatdoost and colleagues [55], comparing the highest category of coffee consumption with the lowest intake was associated with a 7% reduction in the risk of hypertension (RR=0.93, 95% CI, 0.88–0.97). The associations did not differ significantly by age and sex [55].

**Fig. 1:**
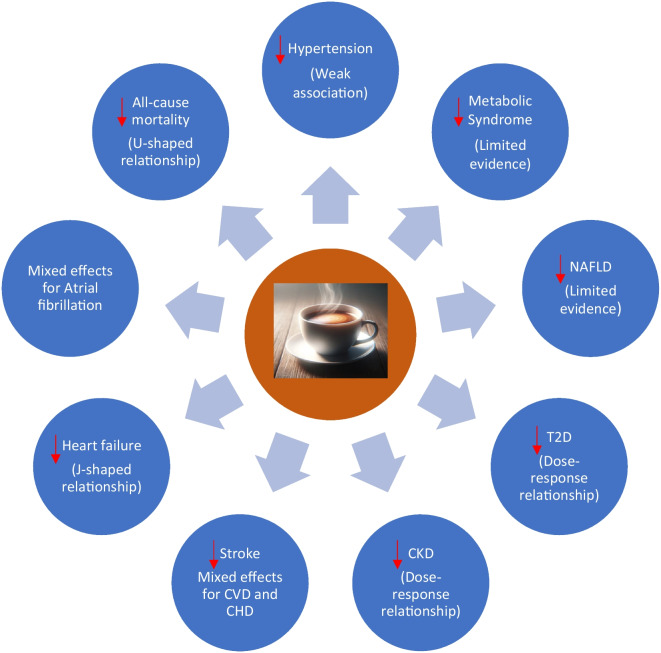
Coffee consumption and adverse cardiometabolic outcomes: summary of effects. AF, atrial fibrillation; BP, blood pressure; CHD, coronary heart disease; CKD, chronic kidney disease; CVD, cardiovascular disease; HF, heart failure; HYPT, hypertension; MetS, metabolic syndrome; NAFLD, nonalcoholic fatty liver disease; T2D, type 2 diabetes

Although the precise nature of the relation between coffee and BP is still unclear, most of evidence suggests that coffee consumption may cause short-term increases in BP, with no effect on long-term BP levels. Furthermore, coffee consumption does not increase the risk of hypertension; a weak association between moderate to high (range 2–8 cups/day) coffee consumption and decreased risk of hypertension cannot be ruled out (Fig. 1), and this does not appear to be modified significantly by age or sex.

### Metabolic syndrome

The relationship between coffee consumption and MetS has mostly been investigated using cross-sectional study designs, with relatively few based on observational prospective cohort studies. Among 93,179 individuals from two large general population cohorts in a MR study, Nordestgaard and colleagues [56] in 2015 showed that coffee intake was associated with a lower risk of MetS observationally. Compared with individuals with no coffee intake, odds ratios (ORs) for MetS were 0.91 (95% CI, 0.86–0.97) for 0.1–1 cup/day, 0.89 (95% CI, 0.84–0.94) for 1.1–2 cups/day, 0.88 (95% CI, 0.83–0.93) for 2.1–3 cups/day, 0.83 (95% CI, 0.78–0.89) for 3.1–4 cups/ day, 0.84 (95% CI, 0.79–0.90) for 4.1–5 cups/day, and 0.89 (95% CI, 0.83–0.95) for >5 cups/day [56]. The inverse associations did not vary significantly by age or sex [56]. Among 2554 older Australian adults followed over a 10-year period, Wong and colleagues [57] in 2022 showed that coffee consumption was not associated with the incidence of MetS. In a cohort of 10,253 participants without MetS at baseline, Corbi-Cobo-Losey and colleagues [58] in 2023 investigated the association between coffee consumption and incident MetS and showed that coffee consumption of ≥1 to <4 cups/day (moderate consumption) was associated with a significantly lower odds of developing MetS compared to consumption of <1 cup/month [58]. Compared with <1 cup/month, ORs were 0.79 (95% CI, 0.53–1.16) for ≥1 cup/month to <1 cup/day, 0.71 (95% CI, 0.50–0.99) for ≥1 cup/day to <4 cups/day, and 0.73 (95% CI, 0.42–1.29) for ≥4 cups/day [58]. There was no significant evidence of interactions by age or sex [58]. In a 2021 meta-analysis that pooled data separately on 13 cross-sectional studies and 2 observational cohort studies, none of the summary estimates showed evidence of an association between coffee consumption and the MetS [59]. In a 2021 systematic review and meta-analysis of RCTs to evaluate the effects of coffee consumption on MetS parameters, Ramli and colleagues [50] showed that green coffee extract supplementation reduced waist circumference, triglyceride levels, HDL-C levels, SBP, and DBP, whereas decaffeinated coffee reduced fasting blood glucose levels.

Limited prospective evidence suggests that moderate to high coffee consumption might be associated with a reduced risk of MetS (Fig. 1).

### Nonalcoholic fatty liver disease

Only few prospective studies have evaluated the association between coffee consumption and the risk of NAFLD; most of the evidence is based on cross-sectional study designs, which lack temporality.

Zelber-Sagi and colleagues [60] prospectively evaluated the association between coffee consumption and onset of NAFLD in the general population and demonstrated no evidence of an association. In a 2017 prospective analysis of a multiethnic cohort, Setiawan and colleagues [61] showed evidence of an inverse association between coffee consumption and the risk of NAFLD, consistent with a dose-response relationship. Compared with individuals with never drinkers, ORs were 1.00 (95% CI, 0.89–1.12) for <1 cup/day, 0.93 (95% CI, 0.84–1.03) for 1 cup/day, 0.85 (95% CI, 0.75–0.96) for 2–3 cups/day, and 0.66 (95% CI, 0.53–0.83) for ≥4 cups/day. Chung and colleagues [62] in 2020 evaluated the association between coffee consumption and fatty liver disease in a large Korean cohort and demonstrated that the incidence of fatty liver was not associated with the amount of coffee consumption at baseline, but was lowered with an increment in the amount of coffee consumption at the follow-up period overall and in males but not in females [62]. Multiple meta-analyses have demonstrated a protective association of coffee intake with the development of NAFLD, but they mostly combined observational cross-sectional, case-control and cohort studies [63–65].

In summary, coffee consumption might be associated with a reduced risk of NAFLD in a dose-response manner, but this is based on limited prospective evidence (Fig. 1).

### Type 2 diabetes

Numerous individual studies have shown that long-term coffee consumption is consistently associated with a significantly lower risk of developing T2D. Using the Nurses’ Health Study (NHS) and Health Professionals’ Follow-up Study (HPFS), Salazar-Martinez and colleagues [66] in 2004 evaluated the long-term relationship between coffee consumption and other caffeinated beverages and the incidence of T2D. Coffee consumption was assessed every 2 to 4 years. Compared to no coffee consumption, the RRs of T2D risk in men were 0.98 (95% CI, 0.84–1.15) for <1 cup/day, 0.93 (95% CI, 0.80–1.08) for 1–3 cups/day, 0.71 (95% CI, 0.53–0.94) for 4–5 cups/day, and 0.46 (95% CI, 0.26–0.82) for ≥6 cups/day. The results were similar for women and were not modified by smoking or body mass index [66]. The associations between decaffeinated coffee and T2D risk were inverse and modest [66]. In a 2006 prospective analysis of the Iowa Women’s Health Study, which included 28,812 postmenopausal women who were free of diabetes and CVD, compared with women who reported 0 cups of coffee/day, women who consumed ≥6 cups/day had a 22% lower risk (RR=0.78; 95% CI, 0.61–1.01) of T2D [67]. This association appeared to be largely driven by decaffeinated coffee (RR=0.67; 95% CI, 0.42–1.08) rather than regular coffee (RR=0.79; 95% CI, 0.59–1.05) [67]. In another analysis of the NHS and HPFS cohorts, Bhupathiraju and colleagues [68] in 2014 examined the associations between 4-year changes in coffee consumption and the risk of T2D in the subsequent 4 years. The results showed that participants who increased their coffee consumption by more than 1 cup/day over a 4-year period had an 11% (95% CI 3%, 18%) lower risk of T2D in the subsequent 4 years compared with those who made no changes in consumption. Furthermore, participants who decreased their coffee intake by more than 1 cup/day had a 17% (95% CI 8%, 26%) higher risk for T2D. In the MR study by Nordestgaard and colleagues [56] in 2015, coffee intake was shown to be associated with a lower risk of T2D observationally. Compared with individuals with no coffee intake, hazard ratios (HRs) for T2D were 0.70 (95% CI, 0.54–0.91) for 0.1–1 cup/day, 0.66 (95% CI, 0.51–0.86) for 1.1–2 cups/day, 0.72 (95% CI, 0.56–0.93) for 2.1–3 cups/day, 0.52 (95% CI, 0.38–0.71) for 3.1–4 cups/day, 0.48 (95% CI, 0.35–0.67) for 4.1–5 cups/day, and 0.57 (95% CI, 0.42–0.78) for >5 cups/day [56]. The inverse associations did not vary significantly by age or sex [56].

Systematic reviews and meta-analysis of these individual studies also support the hypothesis that habitual coffee consumption is linked with a substantially lower risk of T2D. In pooled analysis of nine cohort studies to evaluate the association between habitual coffee consumption and risk of T2D, van Dam and Hu [69] in 2005 reported RRs for T2D to be 0.65 (95% CI, 0.54–0.78) for the highest (≥6 or ≥7 cups/day) and 0.72 (95% CI, 0.62–0.83) for the second highest (4–6 cups/day) category of coffee consumption compared with the lowest consumption category (0 or ≤2 cups/day). The associations did not vary substantially by sex, obesity, or region (USA and Europe) [69]. In a 2014 systematic review and dose-response meta-analysis of 28 prospective studies by Ding and colleagues [70], compared with no or rare coffee consumption, the RR for T2D was 0.92 (95% CI, 0.90–0.94), 0.85 (95% CI, 0.82–0.88), 0.79 (95% CI, 0.75–0.83), 0.75 (95% CI, 0.71–0.80), 0.71 (95% CI, 0.65–0.76), and 0.67 (95% CI, 0.61–0.74) for 1–6 cups/day, respectively. The RR of T2D for a 1 cup/day increase was 0.91 (95% CI, 0.89–0.94) for caffeinated coffee consumption and 0.94 (95% CI, 0.91–0.98) for decaffeinated coffee consumption (*p*-value for interaction = 0.17) [70]. In stratified analyses, the inverse associations between coffee consumption and risk of T2D were similar by geographical region (USA, Europe, and Asia) and sex [70]. In a 2018 meta-analysis involving pooled analysis of 30 prospective studies, the pooled RR was 0.71 (95% CI, 0.67–0.76) for the highest category of coffee consumption (median consumption, 5 cups/day) vs the lowest category (median consumption, 0 cups/day) [36]. The risk of T2D decreased by 6% (RR = 0.94; 95% CI, 0.93–0.95) for each cup/day increase in coffee consumption. Results were similar for caffeinated coffee consumption (per additional cup of coffee per day: RR=0.93; 95% CI, 0.90–0.96) and decaffeinated coffee consumption (RR=0.94; 95% CI, 0.90–0.98) [36]. The data showed no clear differences in the association between coffee consumption and risk of T2D by age, sex, or geographic region [36].

In summary, a significant body of robust research suggests that coffee consumption is inversely associated with the risk of developing T2D in a dose-response manner; with the largest risk reduction observed for high consumption (≥6 cups/day) (Fig. 1).

### Chronic kidney disease

Lew and colleagues [71] in 2018 analyzed data from a prospective cohort of 63,257 Chinese men and women and demonstrated evidence of an association between coffee intake and end-stage renal disease (ESRD). Compared with individuals with no coffee intake or <1 cup/day, HRs were 0.91 (95% CI, 0.79–1.05) for 1 cup/day and 0.82 (95% CI, 0.71–0.96) for ≥2 cups/day. When stratified by sex, this association was observed in men but not in women [71]. Jhee and colleagues [72] in analysis of the Korean Genome and Epidemiology Study (KoGES) cohort in 2018 demonstrated that daily coffee intake was associated with a decreased risk of CKD. Compared with no coffee intake, HRs were 0.76 (95% CI, 0.63–0.92) for 1 cup/day and 0.80 (95% CI, 0.65–0.98) for ≥2 cups/day [72]. In a 2018 analysis of the Atherosclerosis Risk in Communities (ARIC) Study, higher coffee consumption was shown to be associated with a lower risk of CKD [73]. Compared with individuals with no coffee intake, HRs were 0.90 (95% CI, 0.82–0.99) for <1 cup/day, 0.90 (95% CI, 0.82–0.99) for 1 to <2 cups/day, 0.87 (95% CI, 0.77–0.97) for 2 to <3 cups/day, and 0.84 (95% CI, 0.75–0.94) for ≥3 cups/day [73]. The associations were similar in males and females [73]. Srithongkul and Ungprasert [74] in 2020 conducted a meta-analysis of four observational cohort studies and reported a decreased risk of incident CKD among coffee-drinkers compared with non-drinkers: pooled RR of 0.87 (95% CI, 0.81–0.95). In a 2021 meta-analysis of seven prospective cohort studies, coffee consumption was associated with a significant decrease in the risk for incident CKD, consistent with a dose-response relationship. Compared with non-drinkers, the RR of CKD for coffee-drinkers was 0.86 (95% CI, 0.76–0.97); furthermore, compared with non-drinkers, the RR was 0.87 (95% CI, 0.77–0.98) for ≤1 cup/day and 0.82 (95% CI, 0.74–0.92) for ≥2 cups/day [75]. There was no significant evidence that sex modified the association (albeit based on limited number of studies) [75]. In analysis of over 350,000 participants from the UK Biobank, Tang and colleagues [76] in 2022 demonstrated coffee consumption to be associated with a reduced risk of CKD in a dose-dependent manner. Compared with individuals with no coffee intake, HRs were 0.94 (95% CI, 0.88–1.00) for ≤1 cup/day, 0.89 (95% CI, 0.83–0.95) for 2–3 cups/day, 0.86 (95% CI, 0.79–0.94) for 4–5 cups/day, and 0.85 (95% CI, 0.75–0.95) for ≥6 cups/day. Subgroup analysis showed that the inverse coffee-CKD relationship existed in females, but not males. The coffee–CKD association did not significantly differ by age and lifestyle factors such as smoking status and alcohol consumption. Furthermore, the associations did not differ by coffee types (instant, ground, and decaffeinated) [76].

A consistent body of evidence suggests a protective effect of coffee consumption on CKD risk, and this is consistent with a dose-response relationship; higher doses are associated with the largest risk reductions (Fig. 1).

### Cardiovascular disease including coronary heart disease and stroke

#### CHD

The link between coffee consumption and CVD, including CHD and stroke, is an area of ongoing research, with studies yielding mixed results. In a prospective evaluation of 20,179 randomly selected eastern Finnish men and women, Kleemola and colleagues [77] in 2000 showed that coffee consumption was not associated with the risk of nonfatal MI. Lopez-Garcia and colleagues [78] in 2006 evaluated the association between long-term habitual coffee consumption and risk of CHD in the HPFS and NHS, with cumulative coffee consumption categorized as <1 cup/month, 1 cup/month to 4 cups/week, 5 to 7 cups/week, 2 to 3 cups/day, 4 to 5 cups/day, and ≥6 cups/day. The results showed no significant evidence of associations between coffee consumption and CHD in men and women. Grioni and colleagues [79] in 2015 investigated 12,800 men and 30,449 women without a history of CVD and showed that consumption of over 2 cups/day of Italian-style coffee was associated with an increased risk of CHD: HRs of 1.37 (95% CI, 1.03–1.82) for >2–4 cups/day and 1.52 (95% CI 1.11–2.07) for over 4 cups/day.

Sofi and colleagues [37] in their 2007 meta-analysis of 13 case-control and 10 cohort studies showed a significant association between high coffee consumption and increased risk of CHD in the case-control studies: ORs of 1.83 (95% CI, 1.49–2.24) for >4 cups/day and 1.33 (95% CI, 1.04 to 1.71) for 3 to 4 cups/day, with no significant evidence of associations in the long-term follow-up cohort studies. In a 2009 meta-analysis of 21 prospective cohort studies, coffee consumption was not associated with the risk of CHD. Compared to light coffee consumption (<1 cup/day in US or ≤2 cups/day in Europe), the pooled RRs for CHD were 0.96 (95% CI, 0.87–1.06) for moderate (1–3 or 3–4 cups/day), 1.04 (95% CI, 0.92–1.17) for heavy (4–5 or 5–6 cups/day), and 1.07 (95% CI, 0.87–1.32) for very heavy (≥6 or ≥7 cups/day) categories of coffee consumption [80]. However, in subgroup analysis, moderate coffee consumption was associated with reduced risk of CHD in women, but not in men: RRs of 0.82 (95% CI, 0.73–0.92) and 1.01 (95% CI, 0.89–1.14), respectively [80]. In a 2018 meta-analysis of 6 cohort studies and 11 case-control studies, Mo and colleagues [81] showed that compared with <1 cup, daily consumption of 3–4 cups and >4 cups of coffee were significantly associated with an increased risk of MI: pooled ORs were 1.40 (95% CI, 1.11–1.77) and 1.48 (95% CI, 1.22–1.79), respectively. The dose–response relationship was consistent with a “J–shaped” curve; the increased risk of MI was observed in men but not women [81]. However, the associations did not vary by geographical location (Europe and North America) and coffee subtype (caffeinated and decaffeinated) [81]. In a 2023 meta-analysis of 32 prospective cohort studies, comparing the highest category of coffee consumption in comparison with the lowest intake was not associated with the risk of CHD (RR=1.05, 95% CI, 0.97–1.14) [38]. In a subgroup analysis by gender, coffee consumption was associated with an increased risk of CHD in men (RR = 1.19, 95% CI 1.05–1.35), but not in women (RR = 0.91, 95% CI 0.77–1.08) [38].

#### Stroke

In the 24-year follow-up of the NHS, Lopez-Garcia and colleagues [82] in 2009 showed that habitual coffee consumption may modestly reduce risk of stroke: RRs of 0.98 (95% CI, 0.84–1.15) for 1 cup/month to 4 cups/week, 0.88 (95% CI, 0.77–1.02) for 5 to 7 cups/week, 0.81 (95% CI, 0.70–0.95) for 2 to 3 cups/day, and 0.80 (95% CI, 0.64–0.98) for ≥4 cups/day compared to <1 cup/month. These results applied to both ischemic and hemorrhagic stroke and the association was stronger among never and past smokers than among current smokers [82]. The results were qualitatively similar for caffeinated and decaffeinated coffee [82]. In a 2021 analysis of the UK Biobank cohort, Zhang and colleagues [83] demonstrated nonlinear associations of coffee consumption with the risk of stroke; coffee consumption of 2–3 cups/day was associated with the highest risk reduction. Compared to no coffee consumption, the HRs for stroke were 0.90 (95% CI, 0.85–0.95) for 0.5–1 cup/day, 0.88 (95% CI, 0.84–0.94) for 2–3 cups/day, and 0.92 (0.86–0.98) for ≥4 cups/day. The results were qualitatively similar for ischemic and hemorrhagic stroke [83].

In a 2011 dose-response meta-analysis of 11 prospective cohort studies, there was some evidence of a nonlinear association between coffee consumption and risk of stroke [84]. Compared with no coffee consumption, the RRs of stroke were 0.86 (95% CI, 0.78–0.94) for 2 cups/day, 0.83 (95% CI, 0.74–0.92) for 3–4 cups/day, 0.87 (95% CI, 0.77–0.97) for 6 cups/day, and 0.93 (95% CI, 0.79–1.08) for 8 cups/day [84]. The associations were similar for males and females and across geographical regions [84]. In a 2021 meta-analysis of seven long-term cohort studies, comparing the highest versus lowest category of coffee consumption was associated with a reduced risk of overall, hemorrhagic, and ischemic stroke: HRs of 0.92 (95% CI, 0.86–0.99), 0.90 (95% CI, 0.82–0.97), and 0.83 (95% CI, 0.74–0.88), respectively [85]. The results were similar in females [85]. In another 2021 meta-analysis which involved 21 studies including 30 independent cohorts comprising more than 2.4 million participants, findings showed evidence of a significant inverse association between coffee consumption and risk of stroke [86]. The pooled RR for the highest versus the lowest categories of coffee consumption was 0.87 (95% CI, 0.80–0.94). A dose-response analysis was consistent with a nonlinear relationship (U-shape). The strongest association for stroke (21% lower risk) was found for coffee consumption of 3–4 cups/day, with no further reduction in stroke risk observed with increasing levels of coffee consumption beyond this amount [86]. Similar associations were observed for males and females [86].

#### CVD

In 2016, Nordestgaard and Nordestgaard [87] investigated observational and causal associations between coffee intake and CVD mortality among 95,000–223,000 individuals. In observational analyses, CVD mortality appeared to be lower with higher coffee intake [87]. Compared with individuals with no coffee intake, HRs were 0.99 (95% CI, 0.76–1.29) for 0–1 cup/day, 1.04 (95% CI, 0.80–1.36) for 1–2 cups/day, 0.92 (95% CI, 0.70–1.21) for 2–3 cups/day, 0.93 (95% CI, 0.68–1.27) for 3–4 cups/day, 0.71 (95% CI, 0.50–1.00) for 4–5 cups/day, and 0.81 (95% CI, 0.59–1.12) for >5 cups/day. The associations were less prominent in never smokers compared with former and current smokers [87]. In analysis of 347,077 individuals in the UK Biobank, including 8368 incident CVD cases, Zhou and Hyppönen [88] in 2019 showed the association between habitual coffee intake and CVD risk to be nonlinear, and, compared with participants drinking 1–2 cups/day, the risk of CVD was increased for non-drinkers, drinkers of decaffeinated coffee, and those who reported drinking >6 cups/day: ORs of 1.11 (95% CI, 1.04–1.18), 1.07 (95% CI, 1.00–1.15), and 1.22 (95% CI, 1.07–1.40), respectively. There was no evidence of associations for <1 cup/day, 3–4 cups/day, and 5–6 cups/day [88]. In a 2014 meta-analysis of 36 prospective cohort studies comprising 1.2 million participants and over 36,000 CVD cases, a nonlinear relationship between coffee consumption and CVD risk was demonstrated. Moderate coffee consumption was associated with a reduced CVD risk, with the lowest CVD risk at 3 to 5 cups/day, and heavy coffee consumption was not associated with an increased CVD risk [89]. Compared with the lowest category of coffee consumption (median 0 cups/day), the pooled RR for incident CVD was 0.89 (95% CI, 0.84–0.94) for the third highest category (median 1.5 cups/day), 0.85 (95% CI, 0.80–0.90) for the second highest category (median 3.5 cups/day), and 0.95 (95% CI, 0.87–1.03) for the highest category (median 5 cups/day) of coffee consumption. The results were qualitatively similar for CHD and stroke outcomes [89]. In stratified analyses, the results were similar across age, sex, smoking status, geographical location, and coffee subtype (caffeinated and decaffeinated) [89]. In a 2016 dose-response meta-analysis of 31 prospective cohort studies on the association between coffee consumption and CVD mortality risk, with stratified analyses by smoking status and other potential confounders, Grosso and colleagues [90] demonstrated decreased CVD mortality risk (RR=0.85, 95% CI, 0.77–0.93) for consumption of up to 4 cups/day of coffee, with no further decrease in risk for higher consumption. The dose-response relationship was J-shaped for smokers, but linear for non-smokers. The coffee–CVD mortality association did not significantly differ by gender, geographical area, year of publication, and type of coffee [90]. In an updated dose-response meta-analysis of 40 prospective cohort studies, Kim and colleagues [91] in 2019 showed a non-linear inverse association between coffee consumption and CVD mortality. The lowest RR was at 2.5 cups/day for CVD mortality (RR=0.83, 95% CI, 0.80–0.87), with no further increase in risk with additional consumption [91]. In a 2022 analysis of the UK Biobank cohort comprising approximately half a million participants, Chieng and colleagues [35] showed that habitual coffee intake of up to 5 cups/day was associated with significant reductions in the risk of incident CVD and CVD mortality, when compared with non-drinkers. The lowest risk for CHD and ischemic stroke was observed in those who consumed 2–3 cups/day: HRs of 0.89 (95% CI, 0.86–0.91) and 0.84 (95% CI, 0.78–0.90), respectively. All coffee subtypes were associated with a reduction in incident CVD, the lowest risk was 2–3 cups/day for decaffeinated, ground, and instant coffee vs. non-drinkers [35].

Given that coffee consumption may produce short-term increases in blood pressure [51], the impact of coffee consumption on CVD in individuals with hypertension is of interest. Teramoto and colleagues [92] evaluated the impact of coffee consumption on CVD mortality among people with and without hypertension. Coffee consumption was associated with an increased risk of CVD mortality among people with grade 2–3 hypertension; HRs of 0.98 (95% CI, 0.67–1.43) for <1 cup/day, 0.74 (95% CI, 0.37–1.46) for 1 cup/day, and 2.05 (95% CI, 1.17–3.59) for ≥2 cups/day, compared with non–coffee drinkers [92]. There were no significant evidence of associations among people with optimal and normal, high-normal BP, and grade 1 hypertension [92]. In pooled analysis of seven observational cohort studies, there was no evidence of an association between habitual coffee consumption and a higher risk of CVD in individuals with hypertension [51].

In summary, the impact of coffee consumption on heart health remains a subject of debate. Evidence on the association between coffee consumption and CVD is mixed, but a U-shape relationship cannot be ruled out. The overall evidence suggests that coffee consumption is not associated or may be associated with an increased risk of CHD, whereas coffee consumption may be associated with a reduced risk of stroke, with the largest risk reductions observed for moderate consumption (Fig. 1).

### Other cardiovascular outcomes

#### Heart failure

In a 2012 dose-response meta-analysis of five prospective cohort studies of coffee consumption and HF risk, a J-shaped relationship was observed between coffee consumption and HF [93]. Compared with no consumption, the pooled RR for HF was 0.96 (95% CI, 0.90–0.99) for 1–2 servings/day, 0.93 (95% CI, 0.86 to 0.99) for 2–3 servings/day, 0.90 (95% CI, 0.82–0.99) for 3–4 servings/day, 0.89 (95% CI, 0.81–0.99) for 4–5 servings/day, 0.91 (95% CI, 0.83–1.01) for 5–6 servings/day, 0.93 (95% CI, 0.85–1.02) for 6–7 servings/day, 0.95 (95% CI, 0.87–1.05) for 7–8 servings/day, 0.97 (95% CI, 0.89–1.07) for 8–9 servings per day, 0.99 (95% CI, 0.90–1.10) for 9–10 servings/day, 1.01 (95% CI, 0.90–1.14) for 10–11 servings/day, and 1.03 (95% CI, 0.89–1.19) for 11 servings/day. There was no evidence that the relationship between coffee consumption and HF risk varied by sex [93]. In a machine learning analysis of the Framingham Heart Study (FHS), Cardiovascular Heart Study (CHS), and the ARIC study, Stevens and colleagues [94] in 2021 showed that higher coffee intake was associated with reduced risk of HF in all three studies. Compared with no coffee consumption, the HR for HF was 0.69 (95% CI, 0.55–0.87) for 2 cups/day and 0.71 (95% CI, 0.58-0.89) for ≥3 cups/day [94]. In a 2022 analysis of the UK Biobank cohort comprising approximately half a million participants, Chieng and colleagues [35] showed that coffee consumption at all levels was associated with significant reduction in the risk of congestive cardiac failure (CCF). The lowest risk was observed in those who consumed 2–3 cups/day, with HR of 0.83 (95% CI, 0.79–0.87) [35]. All coffee subtypes (decaffeinated, instant, and ground) were associated with a reduction in the risk of CCF [35]. In a 2023 evaluation of the UK Biobank cohort comprising approximately half a million adult men and women, Han and colleagues [95] demonstrated a nonlinear J-shaped association between coffee consumption and HF risk. Compared with drinking coffee <1 cup/day, the HRs for HF were 0.88 (95% CI, 0.84–0.92) for 1–2 cups/day, 0.92 (95% CI, 0.87–0.97) for 3–4 cups/day, and 1.21 (95% CI, 1.06–1.39) for >6 cups/day [95]. Stratified analyses by gender and smoking status yielded similar results, except that >6 cups/day did not significantly increase the risk of HF [95]. The associations were similar for coffee subtypes (decaffeinated, instant and ground) [95].

The overall evidence suggests that coffee consumption is associated with a reduced risk of HF, consistent with a J-shaped relationship. Moderate consumption (range 2–5 cups/day) is associated with the largest risk reduction. Higher consumption may be associated with an increased risk of HF (Fig. 1).

#### Atrial fibrillation and arrhythmias

The link between coffee consumption and AF has been investigated in numerous individual studies as well as pooled analyses of these studies. In a 2014 meta-analysis of six observational cohort studies, coffee/caffeine intake was weakly associated with a reduced risk of AF (RR=0.90; 95% CI, 0.81–1.01) [96]. In subgroup analyses, there was an 11% reduction for low doses (RR=0.89; 95% CI, 0.80–0.99) and 16% for high doses (RR=0.84; 95% CI, 0.75–0.94). Dose-response analysis showed the incidence of AF decreased by 6% (RR=0.94; 95% CI, 0.90–0.99) for every 300 mg/day increment in habitual caffeine intake [96]. In a 2021 meta-analysis of 12 observational cohort studies, caffeine/coffee consumption was not associated with an increased or decreased risk of new-onset AF compared with no caffeine/coffee consumption (pooled RR=0.98; 95% CI, 0.88–1.09) [97]. The highest category of caffeine/coffee consumption (≥5 cups/day) was not associated with an increased or decreased risk of new-onset AF compared with the lowest category (1–2 cups/day) (pooled RR=0.95; 95% CI, 0.84–1.06) [97]. These findings were consistent with previous meta-analyses on the same topic [98–100]. In a 2022 analysis of the UK Biobank cohort comprising approximately half a million participants, Chieng and colleagues [35] demonstrated a U-shaped relationship between increasing levels of coffee consumption and incidence of any arrhythmia (defined as ectopy, AF/flutter, supraventricular tachycardia (SVT), or ventricular tachycardia (VT)/ventricular fibrillation (VF)). The lowest risk for arrhythmias was seen in those who consumed 2–3 coffee cups/day, with a HR of 0.91 (95% CI, 0.88–0.94). For AF/flutter, significant risk reductions were seen in those who consumed between 1 and 5 cups/day, with the peak risk reduction seen in 4–5 cups/day (HR=0.88, 95% CI, 0.83–0.94). For VT/VF, increasing coffee consumption was associated with lower risk of incident arrhythmia, with the lowest risk seen in 4–5 cups/day (HR=0.83, 95% CI 0.70–0.97). In specific evaluation of coffee subtypes, ground and instant coffee consumption was associated with a significant reduction in arrhythmia at 1–5 cups/day but not for decaffeinated coffee. The lowest risk was 4–5 cups/day for ground coffee (HR=0.83, 95% CI, 0.76–0.91) and 2–3 cups/day for instant coffee (HR=0.88, 95% CI, 0.85–0.92) [35]. In a 2022 updated meta-analysis of 10 observational cohort studies, coffee consumption was not associated with the risk of AF: compared with the lowest coffee intake level, the pooled RR for AF was 0.96 (95% CI, 0.88–1.03) for the highest intake (median ≥ 4 cups/day) and 0.93 (95% CI, 0.88–1.03) for the second-highest (median 2.5 cups/day) intake of coffee [101]. In dose-response analysis, the RRs of AF risk estimated directly from the dose–response curve were 1.01 (95% CI, 0.98–1.03), 1.00 (95% CI, 0.97–1.04), 0.99 (95% CI, 0.92–1.02), 0.95 (95% CI, 0.89–1.01), 0.94 (95% CI, 0.87–1.01), 0.89 (95% CI, 0.79–1.02), and 0.87 (95% CI, 0.76–1.02) for 1–7 cups of coffee per day, respectively [101]. There was no significant evidence that sex modified the associations between coffee consumption and AF risk [101]. In a 2023 prospective, randomized, case-crossover trial to examine the effects of caffeinated coffee on cardiac ectopy, arrhythmias, and other outcomes, Marcus and colleagues [102] demonstrated that the consumption of caffeinated coffee did not result in significantly more daily premature atrial contractions than the avoidance of caffeine.

The overall evidence remains mixed, with most of the evidence showing no significant evidence of an association between coffee consumption and risk of AF. However, a weak association between moderate coffee consumption and reduced risk of AF cannot be ruled out (Fig. 1).

### All-cause mortality

The relationship between coffee consumption and all-cause mortality has been extensively studied, with most research indicating a beneficial link. Malerba and colleagues [103] in their 2013 meta-analysis which was based on 23 prospective cohort studies showed that coffee intake was inversely associated with all-cause mortality. The pooled RR of all-cause mortality comparing the highest versus lowest (≤1 cup/day) coffee drinking categories was 0.88 (95 % CI, 0.84–0.93) [103]. Similar associations were observed in males and females [103]. In a 2014 meta-analysis of 20 prospective cohort studies, coffee consumption was shown to be associated with a reduced risk of all-cause mortality, consistent with a nonlinear dose-response relationship [104]. The RR of all-cause mortality comparing high (≥5–9 or ≥2–4 cups/day) vs low/no coffee consumption was 0.86 (95% CI, 0.80–0.92). The pooled RR comparing moderate (1–2 cups/day) vs low/no coffee consumption was 0.92 (95% CI, 0.87–0.98). The inverse association was similar for men and women [104]. In another 2014 meta-analysis which was based on 21 prospective cohort studies, Crippa and colleagues [105] demonstrated strong evidence of nonlinear associations between coffee consumption and all-cause mortality. The largest risk reduction was observed for 4 cups/day: RR of 0.84 (95% CI, 0.82–0.87) [105]. The associations were similar for males and females [105]. In a 2015 analysis of three large ongoing cohort studies (NHS, NHS II, and HPFS), Ding and colleagues [106] demonstrated a nonlinear association between coffee consumption and risk of all-cause mortality, with moderate coffee consumption being associated with lower mortality risk, and high coffee consumption not being associated with mortality risk. Relative to no coffee consumption, the pooled HR was 0.95 (95% CI, 0.91–0.99) for ≤ 1cup/day, 0.91 (95% CI, 0.88–0.95) for 1.1–3 cups/day, 0.93 (95% CI, 0.89–0.97) for 3.1–5 cups/day, and 1.02 (95% CI, 0.96–1.07) for >5 cups per day. Similar results were found for caffeinated and decaffeinated coffee. The association became linear and inverse when analysis was restricted to never smokers [106]. Zhao and colleagues in a 2015 meta-analysis of 17 prospective cohort studies demonstrated a U-shaped dose-response relationship between coffee consumption and all-cause mortality [107]. Compared with non/occasional coffee drinkers, the RRs for all-cause mortality were 0.89 (95% CI, 0.85, 0.93) for 1 to <3 cups/day, 0.87 (95% CI, 0.83, 0.91) for 3 to <5 cups/day, and 0.90 (95% CI 0.87, 0.94) for ≥5 cups/day, and the relationship was more marked in females than in males [107]. Nordestgaard and Nordestgaard [87] in 2016 investigated observational and causal associations between coffee intake and all-cause mortality among 95,000–223,000 individuals. Their observational analyses showed U-shaped associations between coffee intake and all-cause mortality; the lowest risk was observed in individuals with moderate coffee intake (2–5 cups/day) [87]. Compared with individuals with no coffee intake, HRs were 0.87 (95% CI, 0.78–0.96) for 0–1 cup/day, 0.89 (95% CI, 0.79–0.99) for 1–2 cups/day, 0.79 (95% CI, 0.70–0.88) for 2–3 cups/day, 0.87 (95% CI, 0.77–0.99) for 3–4 cups/day, 0.78 (95% CI, 0.68–0.89) for 4–5 cups/day, and 0.81 (95% CI, 0.72-0.93 ) for >5 cups/day [87]. In a 2016 dose-response meta-analysis of 31 prospective cohort studies on the association between coffee consumption and all-cause mortality risk, Grosso and colleagues [90] demonstrated decreased all-cause mortality risk (RR=0.86, 95% CI, 0.82–0.89) for consumption of up to 4 cups/day of coffee, with no further decrease in risk for additional consumption. The dose-response relationship was J-shaped for smokers, but linear for non-smokers. The coffee–CVD mortality association did not significantly differ by gender, geographical area, year of publication, and type of coffee [90]. In an updated dose-response meta-analysis of 40 prospective cohort studies, Kim and colleagues [91] in 2019 showed a non-linear inverse association between coffee consumption and all-cause mortality. The lowest RR was at 3.5 cups/day for all-cause mortality (RR=0.85, 95% CI, 0.82–0.89), with no further increase in risk with additional consumption [91]. The inverse association between coffee consumption and all-cause mortality did not vary by age, overweight status, alcohol drinking, smoking status, and caffeine content of coffee [91]. In another 2019 dose-response meta-analysis of 21 observational cohort studies, a nonlinear association between coffee consumption and all-cause mortality was observed [108]. Compared with no or rare coffee consumption, the RR for all-cause mortality for consumption of 3 cups/day was 0.87 (95% CI, 0.84–0.89). Similar inverse associations were observed for males and females and for caffeinated and decaffeinated coffee [108]. In pooled analysis of 12 prospective cohort studies including 248,050 men and 280,454 women from the Asia Cohort Consortium conducted in China, Japan, Korea, and Singapore, Shin and colleagues [109] in 2022 reported an association between coffee consumption and lower risk of all-cause mortality in men and women. Compared to non-coffee drinkers, the pooled RR of all-cause mortality for men were 0.83 (95% CI, 0.79–0.87) for <1 cup/day, 0.78 (95% CI, 0.73–0.83) for 1 to <3 cups/day, 0.76 (0.67–0.85) for 3 to <5 cups/day, and 0.76 (95% CI, 0.71–0.83) for ≥5 cups/day [109]. The corresponding RRs in women were 0.86 (95% CI, 0.82–0.90) for <1 cup/day, 0.80 (95% CI, 0.72–0.89) for 1 to <3 cups/day, 0.65 (0.54–0.78) for 3 to <5 cups/day, and 0.72 (95% CI, 0.63–0.81) for ≥5 cups/day [109]. In a 2022 analysis of the UK Biobank cohort comprising approximately half a million participants, a significant reduction in all-cause mortality was associated with coffee consumption up to 5 cups/day, with the greatest effect seen with 2–3 cups/day (HR=0.86, 95% CI, 0.83–0.89) [35]. All-cause mortality was significantly reduced for all coffee subtypes, with the greatest risk reduction seen with 2–3 cups/day [35].

In summary, coffee consumption is generally associated with a lower risk of all-cause mortality consistent with a nonlinear U-shape, with the largest risk reduction being observed for moderate consumption (range 1–5 cups/day) (Fig. 1).

## Enhancing the healthspan and increasing longevity

Healthspan refers to the period of one’s life that is spent in good health, free from the chronic diseases and disabilities typically associated with aging [110]. The objective of extending the healthspan is to maximize the years of active, healthy living, rather than merely prolonging life. Common strategies to enhance the healthspan include maintaining a balanced diet, engaging in regular physical activity, managing stress, and avoiding harmful substances. Longevity, on the other hand, is defined as the length of an individual’s life. Increasing longevity means extending the number of years lived, ideally while also enhancing the quality of life in those additional years. The evidence suggests that moderate coffee consumption (typically 1–5 cups per day) may play a protective role against several major cardiometabolic diseases, including T2D and CKD, which are prominent contributors to morbidity and mortality. Additionally, coffee’s potential to prevent stroke and its association with reduced all-cause mortality further supports its role in enhancing healthspan and potentially increasing longevity.

## Evidence from Mendelian randomization studies

Mendelian randomization studies provide valuable insights into the causal relationships between exposures and outcomes. Several MR studies have been conducted to assess the causal effects of coffee consumption on adverse cardiometabolic outcomes (Table 1). These studies have mostly utilized genetic variants demonstrated to be associated with coffee and total caffeine consumption in several Genome Wide Association Studies (GWAS) of European, North American, and South American Populations. These include four variants near the *CYP1A1/2* genes (rs2492297, rs2470893) on chromosome 15 and the *AHR* gene (rs4410790, rs6968865) on chromosome 7 [111, 112]. Evidence on the causal relevance of coffee consumption to T2D risk is mixed. While some MR studies have found evidence of a causal association [113], others have found no evidence [56, 114]. Results from recent MR studies have shown evidence of a causal beneficial effect of coffee consumption on kidney function using outcomes such as CKD and albuminuria [115, 116]. Mendelian randomization studies have not conclusively demonstrated a strong causal link between coffee consumption and the risk of hypertension [117], MetS [56], NAFLD [118, 119], CVD [87], specific cardiovascular outcomes such as stroke and its subtypes [87, 120, 121], HF [121, 122], and AF[121, 123] and all-cause mortality [87]. Mendelian randomization studies of coffee consumption and CHD (ischemic heart disease and coronary artery disease (CAD)) have shown no strong evidence of causal associations [87, 114, 121], except for one recent study which showed that genetically predicted coffee consumption was associated with an increased risk of CAD [124].

**Table 1:** Mendelian randomization studies of coffee Plconsumption and adverse cardiometabolic outcomes

Author, year of publication	Design and approach	Results	Interpretation	Limitations reported
Hypertension				
van Oort, 2020	13/14 SNPs for coffee consumption 2-sample MR method (IVW method used as main method) FinnGen Study (15,870 cases and 74,345 controls) and UK Biobank (54,358 cases and 408,652 controls).	No significant association observed for genetically higher coffee consumption and hypertension. FinnGen (OR=0.91, 95% CI, 0.71–1.18) UK Biobank (OR=1.11, 95% CI, 0.93–1.33) Pooled (OR=1.04, 95% CI, 0.90–1.20)	No evidence supporting a causal relationship between coffee consumption and hypertension	Limited precision as a result of the small variance explained by the genetic instruments; Results less generalizable to populations of non-European ancestry
Metabolic syndrome				
Nordestgaard, 2015	5 SNPs for coffee consumption CGPS and CCHS Summary-level data; IVW DIAGRAM consortium (78,021 participants)	No significant association observed for genetically higher coffee consumption and MetS	No evidence supporting a causal relationship between coffee consumption and MetS	Underpowered IV; pleiotropy; collider bias
NAFLD				
Zhang, 2021	4-SNP and 6-SNP instrument for coffee intake Additional 77-SNP instrument 2-sample MR method Summary-level data; IVW UK Biobank (1122 cases and 399,900 healthy controls) Non-UK Biobank GWAS (91,462) and meta-analysis (121,524)	No significant association observed for genetically higher coffee consumption and NAFLD 4-SNP (OR=0.76, 95% CI 0.51–1.14) 6-SNP (OR=0.77, 95% CI 0.48–1.25)	No evidence supporting a causal relationship between coffee consumption and NAFLD	Underpowered; pleiotropy; SNPs may not be strong and specific markers of coffee intake
Yuan, 2022	12 SNPs for coffee consumption; 2 SNPs for caffeine consumption Summary-level data; IVW GWAS meta-analysis (8434 cases and 770,180 non-cases) GWAS meta-analysis (1483 cases and 17,781 non-cases)	No significant associations observed for genetically predicted coffee and caffeine consumption and NAFLD. Coffee consumption (OR=0.74, 95% CI 0.55–1.00) Caffeine consumption (OR=0.78, 95% CI 0.75–1.00)	Suggestive evidence of causal relationships between coffee and caffeine consumption and NAFLD, but not significant	Underpowered; pleiotropy; heterogeneity in NAFLD definition
T2D				
Nordestgaard, 2015	5 SNPs for coffee consumption CGPS and CCHS Summary-level data; IVW DIAGRAM consortium (78,021 participants)	No significant association observed for genetically higher coffee consumption and T2D	No evidence supporting a causal relationship between coffee consumption and T2D	Underpowered IV; pleiotropy; collider bias
Kwok, 2016	3 SNPs for coffee consumption 2-sample MR method DIAGRAM (34,840 cases and 114,981 controls) CARDIoGRAMplusC4D 1000 genomes-based GWAS	Genetically predicted coffee consumption was not associated with T2D (OR=1.02, 95% CI, 0.76–1.36)	No evidence supporting a causal relationship between coffee consumption and T2D	Confounding; population stratification; results less generalizable to populations of non-European ancestry; cannot rule out non-linear effects of coffee on outcome
Lu, 2023	13 SNPs for coffee consumption 2-sample MR method GWAS for T2D (77,418 cases and 356,122 controls) GWAS for coffee consumption (152,634 individuals)	Genetically predicted higher coffee consumption was associated with lower risk of T2D (OR=0.90; 95% CI, 0.83–0.96)	Genetic evidence supporting an inverse association between coffee consumption and T2D	Results only generalizable to East Asians
CKD				
Kennedy, 2020	25 SNPs for coffee consumption 2-sample MR (IVW method) UK Biobank (227,666 participants) CKDGen Consortium (133,814 participants, 12,385 cases of CKD)	Drinking an extra cup of coffee per day conferred a protective effect against CKD G3-G5 (OR=0.84, 95% CI, 0.72–0.98) and albuminuria (OR=0.81; 95% CI, 0.67–0.97).	Genetic evidence of a beneficial effect of coffee consumption on kidney function	Weak instruments for coffee consumption; pleiotropy
Giontella, 2023	2 SNPs for caffeine intake 2-sample MR (IVW method) GWAS meta-analyses for caffeine intake (>47,000 individuals)	Genetically predicted caffeine intake was associated with an increase in estimated GFR and a low risk of CKD (OR=0.84, 95% CI, 0.75–0.94)	Causal association between higher caffeine intake and low risk of CKD	Findings might not be generalizable to a population of non-European ancestry; Pleiotropy
CVD				
Nordestgaard, 2016	2 SNPs for coffee intake Individual-level data CGPS, CCHS, and CIHDS (112,509 individuals)	Genetically predicted 9% higher coffee consumption was not associated with CVD mortality (HR=1.02, 95% CI, 0.99–1.06)	No evidence supporting a causal relationship between coffee and CVD mortality	Underpowered; pleiotropy; collider bias; confounding by other caffeine-containing beverages; cannot rule out non-linear effects of coffee on outcomes
CHD (IHD and CAD)				
Nordestgaard, 2016	5 SNPs for coffee intake Individual-level data CGPS, CCHS, CIHDS, Cardiogram and C4D (223,414 individuals and 55,689 IHD events)	HR per caffeine intake allele 1.02 (95% CI, 1.00–1.03)	No strong evidence supporting a causal relationship between coffee and IHD	Underpowered; pleiotropy; collider bias; confounding by other caffeine-containing beverages; cannot rule out non-linear effects of coffee on outcomes
Kwok, 2016	3 SNPs for coffee consumption 2-sample MR method DIAGRAM (34,840 cases and 114,981 controls) CARDIoGRAMplusC4D Metabochip/CARDIoGRAM	Genetically predicted coffee consumption was not associated with IHD (OR=0.96, 95% CI, 0.80–1.14)	No evidence supporting a causal relationship between coffee consumption and IHD	Confounding; population stratification; results less generalizable to populations of non-European ancestry; cannot rule out non-linear effects of coffee on outcome
Yuan, 2021	12 SNPs for coffee consumption IVW method Summary-level data UK Biobank (~500,000 participants, 35,979 cases) FinnGen consortium (176,899 participants, 16,631 cases)	50% increase in genetically predicted coffee consumption was not associated with CAD UK Biobank (OR=1.01, 95% CI, 0.85–1.20) FinnGen consortium (OR=1.12, 95% CI, 0.92–1.37)	Limited evidence of a causal relationship between coffee consumption and CAD	Weak instrument bias; model overfitting; inadequate power due to small variance explained by genetic instruments; pleiotropy; cannot rule out non-linear effects of coffee on outcome
Zhang, 2022	14 SNPs for coffee consumption 2-sample MR Summary-level data UK Biobank (~370,000 Europeans) CardiogramplusC4D consortium (60,801 CAD cases and 123,504 controls FinnGen consortium (13,753 cases, 121,885 controls)	Genetically predicted 50% increase of coffee consumption was associated with an increased risk of CAD (OR=1.32, 95% CI, 1.15–1.52) in the fixed-effects model and 1.32 (95% CI, 1.04–1.69) in the random-effects model	Coffee consumption may be causally associated with a higher risk of CAD	Findings might not be generalizable to a population of non-European ancestry
Stroke				
Nordestgaard, 2016	5 SNPs for coffee intake Individual-level data CGPS, CCHS, CIHDS, (112,509 individuals and 4589 ischemic stroke events)	HR per caffeine intake allele 1.02 (95% CI, 0.99–1.02)	No strong evidence supporting a causal relationship between coffee and ischemic stroke	Underpowered; pleiotropy; collider bias; confounding by other caffeine-containing beverages; cannot rule out non-linear effects of coffee on outcomes
Qian, 2020	5 SNPs for coffee intake IVW Summary-level data GWAS (91,462 coffee consumers) MEGASTROKE consortium	Genetically predicted coffee consumption (high vs infrequent/no) was not associated with the risk of stroke or its subtypes: Overall stroke (OR=1.00, 95% CI, 0.94–1.07) ICH (OR=1.06, 95% CI, 0.73–1.55) Ischemic stroke (OR=0.97, 95% CI, 0.90–1.04) Large vessel ischemic stroke (OR=1.05, 95% CI, 0.88–1.26) Cardioembolic ischemic stroke (OR=1.01, 95% CI, 0.86–1.16	Coffee consumption is not causally associated with risk of stroke or its subtypes	Weak instrument bias; pleiotropy; findings might not be generalizable to a population of non-European ancestry; cannot rule out non-linear effects of coffee on outcomes
Yuan, 2021	12 SNPs for coffee consumption IVW method Summary-level data UK Biobank (~500,000 participants, 12,036 stroke cases, 6566 ischemic stroke cases) FinnGen consortium (176,899 participants, 14,171 stroke cases, 8046 ischemic stroke cases)	50% increase in genetically predicted coffee consumption was not associated with stroke and ischemic stroke *Stroke:* UK Biobank (OR=1.04, 95% CI, 0.86–1.26) FinnGen consortium (OR=1.12, 95% CI, 0.91–1.39) *Ischemic stroke:* UK Biobank (OR=0.95, 95% CI, 0.74–1.24) FinnGen consortium (OR=1.14, 95% CI, 0.89–1.47)	Limited evidence of a causal relationship between coffee consumption and stroke	Weak instrument bias; model overfitting; inadequate power due to small variance explained by genetic instruments; pleiotropy; cannot rule out non-linear effects of coffee on outcome
Heart failure				
van Oort, 2020	14 SNPs for coffee consumption 2-sample MR method (IVW method used as main method) GWAS meta-analysis of 26 studies (47,309 cases and 930,014 controls)	No significant association observed for genetically predicted 50% change in coffee consumption and HF (OR=1.06, 95% CI, 0.86–1.31)	No evidence supporting a causal relationship between coffee consumption and HF	Cannot rule out non-linear effects of coffee on outcome; limited precision as a result of the small variance explained by the genetic instruments; results less generalizable to populations of non-European ancestry; survival bias
Yuan, 2021	12 SNPs for coffee consumption IVW method Summary-level data UK Biobank (~500,000 participants, 10,560 cases) FinnGen consortium (176,899 participants, 9576 cases)	50% increase in genetically predicted coffee consumption was not associated with HF UK Biobank (OR=0.97, 95% CI, 0.79–1.18) FinnGen consortium (OR=0.87, 95% CI, 0.67–1.12)	Limited evidence of a causal relationship between coffee consumption and HF	Weak instrument bias; model overfitting; inadequate power due to small variance explained by genetic instruments; pleiotropy; cannot rule out non-linear effects of coffee on outcome
Atrial fibrillation				
Yuan, 2019	9 SNPs for coffee consumption IVW method Summary-level data Atrial Fibrillation Consortium (65,446 cases and 522,744 non-cases) GWAS (375,833 individuals)	50% increase in genetically predicted coffee consumption was not associated with AF (OR=0.98, 95% CI, 0.88–1.10)	Findings do not support a causal association between habitual coffee consumption and risk of AF	Cannot rule out non-linear effects of coffee on outcome; population stratification
Yuan, 2021	12 SNPs for coffee consumption IVW method Summary-level data UK Biobank (~500,000 participants, 23,882 cases) FinnGen consortium (176,899 participants, 17,325 cases)	50% increase in genetically predicted coffee consumption was not associated with AF UK Biobank (OR=1.05, 95% CI, 0.88–1.25) FinnGen consortium (OR=1.00, 95% CI, 0.79–1.27)	Limited evidence of a causal relationship between coffee consumption and AF	Weak instrument bias; model overfitting; inadequate power due to small variance explained by genetic instruments; pleiotropy; cannot rule out non-linear effects of coffee on outcome
All-cause mortality				
Nordestgaard, 2016	2 SNPs for coffee intake Individual-level data CGPS, CCHS and CIHDS (112,509 individuals, 12,656 all-cause mortality events)	HR per caffeine intake allele 1.01 (95% CI, 0.99–1.03)	No evidence supporting a causal relationship between coffee consumption and all-cause mortality	Underpowered; pleiotropy; collider bias; confounding by other caffeine-containing beverages; cannot rule out non-linear effects of coffee on outcomes

*AF* atrial fibrillation, *CAD* coronary artery disease, *CCHS* Copenhagen City Heart Study, *CGPS* Copenhagen General Population Study, *CHD* coronary heart disease, *CKD* chronic kidney disease, *CI* confidence interval, *CIHDS* Copenhagen Ischaemic Heart Disease Study, *CVD* cardiovascular disease, *GWAS* genome-wide association study, *HF* heart failure, *HR* hazard ratio, *ICH* intracerebral hemorrhage, *IHD* ischemic heart disease, *IV* instrumental variable, *IVW* inverse-variance weighted, *MetS* metabolic syndrome, *MR* Mendelian randomization, *NAFLD* nonalcoholic fatty liver disease, *OR* odds ratio, *SNPs* single-nucleotide polymorphisms, *T2D* type 2 diabetes

In summary, whiles some MR studies indicate that coffee consumption may have a protective effect against certain cardiometabolic diseases such as T2D and CKD, the evidence is less clear for other adverse cardiometabolic conditions. The overall impact of coffee on cardiometabolic health appears to be complex and influenced by various factors.

## Potential pathways underlying the cardiometabolic effects of coffee consumption and its bioactive components

**Fig. 2:**
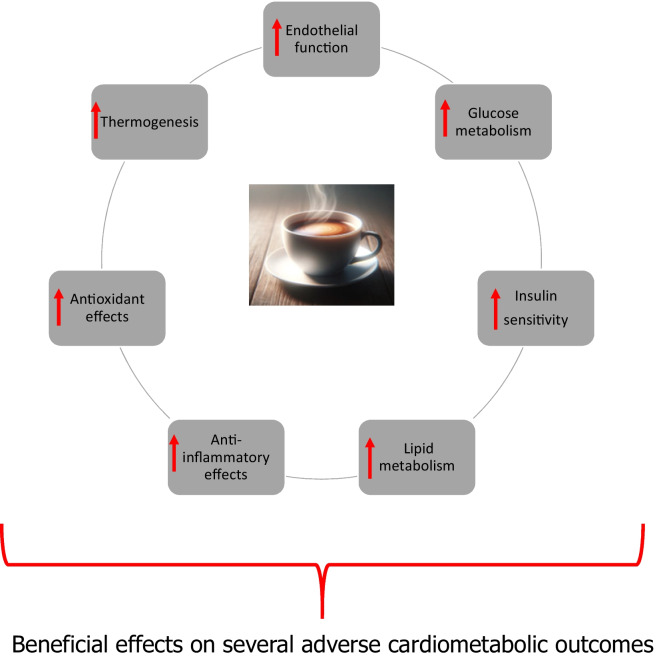
Proposed mechanistic pathways underlying the beneficial effects of coffee consumption on adverse cardiometabolic outcomes

The beneficial effects of coffee on cardiometabolic health are multifaceted, involving a complex interplay of antioxidative, anti-inflammatory, lipid-modulating, insulin-sensitizing, and thermogenic effects (Fig. 2). These mechanisms collectively contribute to reducing the risk of a spectrum of adverse cardiometabolic outcomes, including hypertension, MetS, NAFLD, T2D, CKD, CVDs, and all-cause mortality.

Coffee is rich in numerous bioactive components that are proposed to exert these favorable cardiometabolic effects [125, 126]. Caffeine and its methylxanthine metabolites are known to modulate oxidative stress and inflammation [127], which are pathways involved in the genesis of many cardiometabolic disorders. Polyphenols such as chlorogenic acid and phytic acid also combat oxidative stress and inflammation [128], key factors in the development of CVDs and T2D.

Several polyphenols found in coffee or as metabolites of coffee compounds play significant roles in glucose homeostasis and the health complications associated with glucose dysregulation. These polyphenols include enterodiol, enterolactone, matairesinol, secoisolariciresinol, kaempferol, quercetin, and chlorogenic acid [129]. Enterodiol and enterolactone are lignans metabolized from precursors in coffee by intestinal bacteria and have been studied for their potential in modulating blood glucose levels and improving insulin sensitivity [130, 131]. Similarly, matairesinol and secoisolariciresinol contribute to these lignans’ profiles [131–135], enhancing their effects on metabolic health. Kaempferol and quercetin, both flavonoids, are known for their antioxidant properties, which can mitigate oxidative stress, a key contributor to the pathogenesis of diabetes and its complications [136–140]. These compounds can influence glucose metabolism by modulating signaling pathways involved in insulin signaling and glucose uptake in cells, thereby helping to stabilize blood glucose levels. Chlorogenic acid, one of the most abundant polyphenols in coffee, has a direct impact on glucose metabolism. It inhibits the activity of glucose-6-phosphatase [141–143], an enzyme involved in the release of glucose into the bloodstream, and enhances the performance of insulin, thereby improving glucose uptake in tissues. Chlorogenic acid also modulates gut hormones that regulate glucose and satiety, further aiding in glucose management [144, 145]. In particular, chlorogenic acid and trigonelline have been shown to enhance insulin sensitivity, reduce intestinal absorption of glucose, improve glucose tolerance and metabolism, inhibit gut incretin hormones, and enhance lipid metabolism [146–154], thereby reducing levels of glucose and lipids, consequently lowering the risk or delaying the onset of T2D, MetS, NAFLD, and CVD. The collective impact of these polyphenols on glucose homeostasis makes coffee a significant dietary component in managing and potentially preventing complications associated with glucose dysregulation such as T2D. Their mechanisms of action include anti-inflammatory effects, enhancement of insulin action, modulation of glucose transport, and overall antioxidant protection, all of which are relevant for maintaining cardiometabolic health.

Caffeine promotes lipolysis through phosphodiesterase inhibition, which increases cyclic adenosine monophosphate (cAMP) levels and activates β-adrenergic receptors, stimulating the breakdown of fats [155, 156]. Caffeine regulates fat metabolism via the sympathetic nervous system, promoting the secretion of catecholamines that activates β-adrenergic receptors and downstream pathways for lipid metabolism [156, 157]. Glycochenodeoxycholate, a metabolite of coffee consumption and a lipid involved in primary bile acid metabolism, may contribute to the favorable kidney health outcomes associated with coffee consumption [158]. Coffee is a risk source of minerals and trace elements—it has been reported that 5 cups of coffee/day contribute to approximately 26% of the daily intake of potassium, 12% of the daily intake of magnesium, 10% of the daily intake of manganese, and 15% of the daily intake of niacin [159]. Magnesium for instance may explain some of the beneficial effects of coffee intake on T2D via its positive effects on carbohydrate metabolism [160, 161]. It has been suggested that coffee consumption might reduce the risk of metabolic conditions such as T2D via stimulation of thermogenesis and induction of weight loss [162].

The evidence also suggests that caffeine is likely to be the main ingredient that contributes to the thermogenic effects of coffee, but there is limited evidence from human studies [162]; caffeine has been shown to increase thermogenesis of brown adipose tissue partly by upregulating the expression of an uncoupling protein in rodents [163]. While the acute effects of coffee can temporarily increase BP [51], long-term consumption has been linked to a neutral or beneficial effect on BP [51], potentially producing no adverse impact on hypertension and CVD. A number of proposed mechanisms for the acute BP raising effect of coffee include sympathetic overactivation, antagonism of adenosine receptors, increased norepinephrine release via direct effects on the adrenal medulla, renal effects, and activation of the renin–angiotensin system [164, 165]. Coffee consumption via caffeine potentially lowers BP through enhanced endothelium-dependent vasodilation [166]. Coffee consumption may also exert its favorable cardiometabolic effects via improvement in endothelial function and arterial stiffness [167, 168]. The consistent reduction in the risk of all-cause mortality could be due to the comprehensive effects of coffee on various aspects of cardiometabolic health, including reduced inflammation and oxidation, improved insulin sensitivity, and better lipid profiles [146]. Conversely, phenotypic and genetic evidence suggests that long-term heavy coffee consumption is associated with increased levels of lipids—LDL-C, ApoB, and total-C, with the highest lipid levels seen among participants reported drinking >6 cups/day [169]. In a meta-analysis of 14 RCTs of the effects of coffee consumption on serum lipids, drinking 6 cups/day was significantly associated with an increase in levels of total cholesterol, LDL-C, and triglycerides, but not HDL-C [170]. These results appear to be driven by trials of unfiltered or boiled coffee; furthermore, the increases in levels of serum lipids were greater in individuals with hyperlipidemia [170]. These findings were replicated in another meta-analysis of 12 RCTs [171].

Exosomes are small extracellular vesicles that play a crucial role in intercellular communication. They are involved in various physiological processes, including inflammation, immune response, and tissue repair [172]. Recent studies have highlighted their significance in cardioprotection, demonstrating that exosomes can convey protective signals to cardiac cells, thereby mitigating damage and promoting repair [173–176]. Bioactive compounds found in coffee, such as caffeine and chlorogenic acids, which have been shown to have anti-inflammatory, antioxidant, and cardioprotective effects, can influence the release and composition of exosomes [177, 178], enhancing their adaptive functions. The adaptive exosomes released in response to coffee’s bioactive compounds can carry a variety of protective molecules, including microRNAs (miRNAs), proteins, and lipids [179], which may play a critical role in mediating the protective effects of coffee on cardiovascular health and potentially other organs.

Boiled or unfiltered coffee has a rich diterpene content (namely cafestol and kahweol), which inhibits bile acid synthesis and negatively affects lipid metabolism, making it atherogenic [33, 171, 180]. On the contrary, filtered coffee does not contain diterpene and may exert antiatherogenic effects via increase in HDL-mediated cholesterol efflux from macrophages through the influence of plasma phenolic acid [33]. This unfavourable lipid profile may potentially increase the risk of cardiovascular outcomes, as observed in some studies [88, 181]. However, it has been reported that variations in CYP1A2 activity among coffee consumers rather determines the risk of CVD and not the diterpene content. The caffeine in coffee is metabolized by the polymorphic cytochrome P450 1A2 (CYP1A2) enzyme; CYP1A2 accounts for approximately 95% of caffeine metabolism. Individuals who are homozygous for the CYP1A2*1A allele are “rapid” caffeine metabolizers, whereas carriers of the variant CYP1A2*1F are “slow” caffeine metabolizers [181]. Cornelis and colleagues [181] in their study which sought to determine whether the CYP1A2 genotype modifies the association between coffee consumption and risk of acute nonfatal MI showed that coffee consumption was associated with an increased risk of nonfatal MI only among individuals with slow caffeine metabolism. When that analysis was limited to only individuals who consumed filtered coffee, the association between coffee consumption and increased risk of acute nonfatal MI remained consistent. The findings by Cornelis and colleagues [181] were, however, not replicated by Zhou and Hyppönen [88]. It has also been suggested that the conflicting associations between coffee consumption and CVD may be due to the confounding or effect-modifying effects of smoking as well as the fact that smokers metabolize caffeine more rapidly than nonsmokers due to the well-known CYP1A2-inducing effect of smoking [182]. Some studies have shown that the associations are less prominent in never smokers compared with former and current smokers, others have shown stronger associations among never and past smokers than among current smokers, and still others have shown similar associations among never, former and current smokers [82, 87, 89, 181]. These observations suggest that the pathways underlying the effects of coffee consumption on cardiovascular outcomes are more complex than originally thought. It has been reported the CYP1A2 genotype may modify the association between coffee intake and kidney disease; caffeinated coffee intake has been shown to be associated with an increase in the risk of kidney disease in slow metabolizers but not fast metabolizers [183].

Bioactive compounds in coffee, such as polyphenols, flavonoids, and alkaloids, have been shown to exert significant epigenetic effects that can contribute to cardioprotection. These compounds can influence gene expression through several mechanisms, including DNA methylation, histone modifications, and non-coding RNA (ncRNA) expression [184]. Bioactive compounds, such as chlorogenic acid and caffeic acid, have been shown to modulate DNA methylation patterns [184–186]. These modifications can influence the expression of genes involved in inflammatory pathways, lipid metabolism, and oxidative stress response, which are critical for maintaining cardiovascular health. Coffee components like trigonelline and kahweol have been found to induce histone modifications such as acetylation and methylation [184]. These histone modifications can activate or repress the transcription of genes involved in cellular processes that protect against cardiometabolic diseases. Compounds in coffee can alter the expression of specific ncRNAs, including miRNAs and long non-coding RNAs (lncRNAs) [187] that regulate pathways linked to inflammation, oxidative stress, endothelial function, cell proliferation, and apoptosis. The epigenetic modifications induced by coffee consumption have the potential to exert long-lasting impacts on the epigenome of vital organs, contributing to the maintenance of cardiovascular health.

While most studies have demonstrated similar associations between coffee consumption and adverse cardiometabolic outcomes in males and females, others have shown disparities, especially for the outcome of CKD [71, 76]. This potentially reflects the sex disparity in the pathogenesis of CKD. It has been suggested that sex hormones such as testosterone and sex hormone-binding globulin (SHBG) may partly account for the sex disparities in the associations; the reno-protective effect of coffee appears to be more evident in individuals with higher SHBG and lower testosterone concentrations [76].

Acrylamide is a chemical compound that forms in some foods during high-temperature cooking processes, such as roasting, frying, and baking [188]. In coffee, acrylamide is primarily formed during the roasting process. Different coffee types, such as instant coffee, espresso, and filter coffee, contain varying levels of acrylamide, with instant coffee generally having higher levels compared to espresso and filter coffee due to the differences in roasting and processing methods [189]. Acrylamide has been shown to be both neurotoxic and carcinogenic in animal studies. It has been linked to an increased risk of cancer and damage to the nervous system [190, 191]. However, human epidemiological studies have revealed a general lack of association between dietary acrylamide exposure and the incidence of cancer [188, 192]. The World Health Organization (WHO) and the Food and Agriculture Organization (FAO) have acknowledged the potential risks but also highlight the need for more research to fully understand the implications for human health [193]. From a cardiometabolic perspective, acrylamide’s effects are less clear. While there is evidence that acrylamide exposure can influence metabolic pathways and potentially contribute to adverse cardiovascular outcomes [194], the overall impact of dietary acrylamide, particularly from coffee consumption, remains inconclusive. Some studies suggest that the beneficial compounds in coffee, such as antioxidants, may counteract the potential harmful effects of acrylamide [188], but further research is needed to clarify these interactions.

## Coffee consumption and its bioactive components: impacts on cellular and molecular mechanisms of aging

Coffee consumption may support longevity and healthspan through its effects on fundamental biological processes involved in aging. These include mitigating oxidative stress and inflammation, improving mitochondrial function, enhancing DNA repair, stimulating autophagy, modulating epigenetic regulation, and regulating cellular metabolic pathways. Each of these mechanisms plays a critical role in decelerating the aging process and reducing the incidence of age-related diseases [195].

Research using invertebrate models, such as *Caenorhabditis elegans* [196–198] and *Drosophila melanogaster* [199, 200], provided valuable insights into the potential anti-aging and lifespan-extending effects of coffee and its components. These studies highlighted fundamental biological mechanisms that might also be relevant in higher organisms, including humans. Importantly, there are studies showing that caffeine can extend lifespan in *C. elegans* by influencing cellular stress pathways and metabolism [201–209]. Of note, there are also studies showing no extension of lifespan in fruit flies reared on food containing caffeine [210]. Research also has been conducted on various coffee polyphenols like chlorogenic acid and their impact on aging in invertebrates. These studies predominantly focused on antioxidant and anti-inflammatory properties that could contribute to lifespan extension. For example, chlorogenic acid has been shown to improve stress resistance and extend lifespan in *C. elegans* [196–198] and *D. melanogaster* [199, 200]. Additionally, recent studies provided evidence that coffee compounds, particularly flavonoids, also promote longevity in *Saccharomyces cerevisiae* likely by attenuating oxidative stress [211].

Oxidative stress is also a major contributor to cellular aging and the development of age-related diseases such as cardiometabolic diseases in vertebrates including humans [212–219]. Antioxidants, such as chlorogenic acids, present in coffee can help reduce oxidative stress in the body [128, 220], thereby attenuating cellular aging processes and interfering with the pathogenesis of age-related diseases [221–226].

Nuclear factor erythroid 2–related factor 2 (Nrf2) is a critical transcription factor that plays a central role in cellular defense against oxidative stress and is an essential regulator of the cellular aging process [212, 216, 227–232]. Nrf2 regulates the expression of antioxidant proteins that protect against oxidative damage triggered by injury and inflammation, which are common contributors to the aging process. As organisms age, the efficiency of this protective response can diminish, leading to an increased buildup of oxidative damage and cellular senescence [212, 216, 228]. Importantly, chlorogenic acid, a polyphenol abundant in coffee, has been shown to positively influence the Nrf2 pathway. Research indicates that chlorogenic acid can activate Nrf2 [225], leading to an enhanced transcriptional activity of antioxidant response element (ARE)–driven genes. This activation increases the production of endogenous antioxidant enzymes such as heme oxygenase-1 (HO-1), NAD(P)H quinone oxidoreductase 1 (NQO1), and glutathione S-transferase (GST). These enzymes play pivotal roles in detoxifying reactive oxidants and thus maintaining cellular redox balance. By stimulating the Nrf2 pathway, chlorogenic acid helps fortify cellular homeostatic defense mechanisms against oxidative stressors, potentially mitigating the effects of aging and reducing the risk of age-related diseases. This mechanism underscores the therapeutic potential of dietary components like chlorogenic acid in promoting longevity and enhancing healthspan through modulation of critical aging-related biochemical pathways.

In addition to its antioxidative capabilities, caffeine and its methylxanthine metabolites possess anti-inflammatory effects [127]. Chronic inflammation is a hallmark of aging and is closely associated with the progression of many age-related diseases [195, 213, 233–239]. By reducing inflammation, coffee can help maintain cellular health and improve overall longevity.

Coffee has been shown to boost cellular DNA repair mechanisms [240–244]. Caffeine, in particular, supports the preservation of genomic integrity by enhancing the repair of DNA damage [245], which accumulates with age and contributes significantly to the aging process and the onset of age-related diseases [246]. Telomeres are protective caps on the ends of chromosomes that shorten with each cell division, and their length is an indicator of cellular aging [247] and linked to a variety of aging-related disorders, such as T2D and CVD [248, 249]. Some studies have indicated that higher coffee consumption could be associated with longer telomeres [250], suggesting a potential protective effect against accelerated aging.

Coffee contains various bioactive compounds that influence the expression and activity of sirtuin-1 (SIRT1) [251], a protein that plays a crucial role in cellular regulation, aging, and cardiometabolic health. SIRT1 is a NAD+-dependent deacetylase involved in numerous cellular processes, including DNA repair, inflammation regulation, and mitochondrial function [252]. Polyphenols such as chlorogenic acid have been shown to enhance SIRT1 expression and activity [253]. SIRT1 activation by coffee compounds contributes to longevity by improving mitochondrial function, reducing oxidative stress, maintaining cellular homeostasis, and delaying the onset of age-related diseases [254–261]. Enhanced SIRT1 activity can lower blood lipids, glucose levels, and inflammation [262, 263], thus reducing the risk of CVDs and T2D. SIRT1’s role in deacetylating key transcription factors and enzymes involved in metabolic regulation underscores its importance in maintaining cardiometabolic health.

Coffee and its constituents can stimulate autophagy [264], a process essential for removing damaged cellular components. By enhancing autophagy, coffee helps in maintaining cellular function and longevity. This process is necessary for preventing the buildup of cellular waste that can contribute to aging and related disorders [265]. Coffee influences several metabolic pathways that are linked to aging and metabolic health. It affects lipid metabolism, glucose metabolism, and insulin sensitivity [146–151], which are vital for preventing metabolic diseases, common age-related conditions. The caffeine in coffee has been shown to improve energy metabolism and increase caloric expenditure [163], which can delay the onset of metabolic decline associated with aging.

## Adverse effects of coffee consumption

While coffee consumption is associated with numerous cardiometabolic health benefits, excessive intake can lead to several adverse effects. The most commonly reported negative effects are linked to its main active ingredient, caffeine, which can affect various aspects of health and well-being. Individuals vary greatly in their sensitivity to caffeine. Caffeine stimulates the nervous system causing the release of adrenaline, leading to rapid or irregular heartbeat and temporary spikes in blood pressure [266]. Some may experience jitteriness or palpitations even with small amounts of coffee. High levels of caffeine in coffee can exacerbate feelings of anxiety. Coffee can also significantly disrupt sleep patterns, leading to insomnia, particularly if consumed in the afternoon or evening. Caffeine’s stimulatory effect can delay the onset of sleep and reduce sleep quality [267–270]. Excessive coffee consumption can lead to digestive discomfort in some individuals. Coffee stimulates gastric acid production, which can exacerbate gastrointestinal conditions such as gastroesophageal reflux disease (GERD) and ulcers. It may also cause symptoms like stomach upset and exacerbate irritable bowel syndrome [271]. Rarely, excessive coffee intake can lead to rhabdomyolysis [272], a serious condition in which muscle fibers break down and enter the bloodstream, potentially leading to kidney damage.

High caffeine intake has been linked to reduced calcium absorption, which could potentially lead to bone thinning and osteoporosis. However, the evidence surrounding this association remains controversial [273, 274]. Many studies actually suggest that consumption of coffee is beneficial for bone health [275–277].

Regular, heavy use of caffeine can lead to physical dependence [278]. Caffeine withdrawal can trigger symptoms like headache, fatigue, irritability, and difficulty concentrating. Caffeine can cross the placental barrier during pregnancy and may cause spontaneous abortion and impaired fetal growth [279]. It is recommended that caffeine intake for women who plan to become pregnant and or who are pregnant should not exceed 300mg/day [280].

## Clinical and public health implications

The findings from various studies on coffee consumption and its impact on cardiometabolic outcomes have significant clinical and public health implications. The evidence indicates that while coffee may cause short-term increases in BP, it does not adversely affect long-term BP levels or increase hypertension risk. The weak association with decreased hypertension risk suggests that coffee consumption should not be a primary concern in hypertension management. The suggestion of a reduced risk of MetS with moderate to high coffee consumption, despite limited evidence, highlights a potential area for public health intervention. Further research may validate coffee as a simple dietary intervention to mitigate MetS risk. The strong inverse association between coffee consumption and T2D risk, especially with higher consumption levels, is highly relevant for diabetes prevention strategies. Public health initiatives might consider incorporating coffee consumption as part of lifestyle modification recommendations. The protective effect of coffee against CKD, particularly at higher doses, indicates potential renal benefits of coffee consumption. This could influence dietary advice given to individuals at risk of or managing CKD. The mixed evidence regarding coffee’s impact on heart health, particularly its association with reduced stroke risk but uncertain effects on CHD, highlights the need for individualized dietary recommendations based on personal CVD risk profiles. The J-shaped relationship between coffee consumption and HF risk, with moderate intake offering the most benefit, suggests that moderate coffee consumption could be a simple, accessible measure to reduce HF risk. The mixed evidence on coffee’s impact on AF risk indicates that moderate coffee consumption is unlikely to significantly affect AF risk. This information can reassure patients and clinicians regarding coffee consumption in the context of heart rhythm disorders. The findings on cardiovascular outcomes appear to reflect recommendations in the 2021 European Society of Cardiology guidelines which indicate that coffee consumption of 3–4 cups/day may be moderately beneficial in the prevention of CVD [281]. The general association of coffee consumption with lower all-cause mortality, particularly at moderate levels, supports the inclusion of coffee in a healthy diet. This could be an important consideration in public health guidelines and dietary recommendations. The findings that inverse associations between coffee consumption and adverse cardiometabolic outcomes are generally consistent across different age groups, sexes, geographical regions, and coffee types (instant, ground, decaffeinated) carry relevant implications. These suggest that the health benefits of coffee could be broadly applicable, making coffee a universally beneficial component in dietary guidelines aimed at preventing cardiometabolic conditions. This broad applicability across demographic groups can simplify public health messages and dietary recommendations. The consistency of these health benefits across various coffee types, including decaffeinated coffee, opens the door for a wider population to benefit from coffee consumption, including individuals who are sensitive to caffeine or have specific health concerns like hypertension or anxiety disorders. Clinicians may recommend moderate coffee consumption as part of a healthy lifestyle for most individuals, regardless of their age or sex, knowing that the potential benefits are not significantly influenced by these demographic factors. Given the lack of significant variation in benefits between coffee types, the focus shifts to the quantity of consumption. The importance of considering the method of coffee preparation also needs to be taken into consideration. Boiled or unfiltered coffee, due to its high diterpene content, may pose a risk for cardiovascular health by increasing atherogenic lipids. This suggests that individuals with or at risk for CVD particularly those with dyslipidemia might need to be cautious about their choice of coffee preparation method. Filtered coffee, which lacks diterpenes, could be a healthier alternative. Its potential antiatherogenic effects may make it a more suitable option for those concerned about cardiovascular health, including individuals with a history of heart diseases or elevated lipid levels. Findings from MR studies reinforce the potential protective effects of coffee consumption against specific diseases such as T2D and CKD, highlighting the importance of inclusion of coffee consumption in these disease specific guidelines. The overall evidence suggests that moderate coffee consumption (range of 1–5 cups/day) is generally beneficial or neutral for various cardiometabolic outcomes. By potentially mitigating the risk factors associated with common age-related diseases such as cardiometabolic diseases, regular, moderate coffee consumption could be a valuable component of strategies aimed at extending the healthspan and increasing longevity. This aligns with the broader goal of not only living longer but also living healthier.

## Gaps and future directions

Future research directions in the context of coffee consumption and cardiometabolic outcomes should address several critical areas. Though a number of studies incorporated repeated assessments of coffee consumption over time in their analysis, rather than relying solely on baseline data, more studies adopting this approach are needed. This approach will help minimize regression dilution bias and provide a more accurate picture of coffee consumption patterns and their long-term health impacts. However, it should be acknowledged that coffee consumption is one of the most reproducible dietary items and therefore barely changes over time [282]. For outcomes like hypertension, MetS, NAFLD, CVD, CHD, and AF, where evidence remains limited, inconsistent, and sometimes weak, further large-scale longitudinal studies are required. These studies should aim to clarify the extent of the beneficial associations of coffee consumption with these conditions. Given the variability in defining moderate coffee consumption across studies (ranging from 1 to 5 cups/day), future research should focus on establishing a more precise definition and optimal levels of coffee consumption. This involves investigating the detailed dose-response relationships to determine the optimal amount and frequency of coffee intake for maximum health benefits. Though studies generally suggest that the inverse associations of coffee consumption with adverse cardiometabolic outcomes do not vary substantially across different age groups, sexes, and coffee subtypes, the evidence is still limited and inconsistent in some instances. Future studies should explore these specific associations to understand how coffee consumption impacts diverse populations and to identify any unique effects of different types of coffee. Given the potential influence of the method of coffee preparation (boiled or unfiltered vs filtered) on lipid levels and subsequently on cardiovascular outcomes, additional research is warranted to understand the extent of the impact of diterpenes in boiled/unfiltered coffee on long-term cardiovascular health. This could also include investigating whether certain populations may be more affected by the lipid-raising effects of these coffee types. Apart from T2D and CKD, it appears observational studies showing evidence of inverse associations between coffee consumption and other adverse cardiometabolic outcomes may be confounded by diet and lifestyle factors associated with coffee consumption. These include factors such as smoking, excessive alcohol consumption, poor diet, and a sedentary lifestyle. However, it has been argued that the confounding effects of these variables would tend to bias the results toward positive and not inverse associations [66]. Larger-scale studies are needed to investigate in more detail the confounding and effect-modifying effects (restricting analysis to smokers or never smokers alone) of smoking and other lifestyle factors, which are major risk factors for these adverse cardiometabolic outcomes. A recent MR study indicated evidence that coffee consumption might be causally associated with an increased risk of CAD (CHD) [124], findings which are consistent with some observational studies [37, 79]. Given the inconsistencies and likely limitations of observational studies, additional and adequately powered MR studies are warranted to help determine if coffee consumption is a causal therapeutic target for these cardiometabolic conditions, providing a genetic perspective to the observed associations. It should be acknowledged that MR studies on coffee consumption and outcomes have major shortcomings of relying on gene loci (*CYP1A1/2* and *AHR* gene regions) with major pleiotropic effects [283, 284]. Therefore, all MR assumptions may not hold, which may potentially yield biased causal estimates. Large-scale GWAS are needed to uncover specific genetic determinants of caffeine and coffee consumption. Understanding the biological mechanisms through which coffee exerts its effects is essential. Mechanistic studies should explore the pathways and processes by which coffee consumption influences various cardiometabolic outcomes. Such studies will not only provide scientific insights but may also lead to the development of targeted therapies and interventions. These future directions will not only deepen our understanding of the impact of coffee consumption on health but also inform public health guidelines and clinical practice, ensuring that recommendations regarding coffee consumption are grounded in robust scientific evidence.

## Conclusions

The current body of evidence on coffee consumption and its relationship with various cardiometabolic outcomes presents a complex but largely positive picture. While coffee may cause short-term increases in BP, its long-term consumption does not seem to adversely affect BP and may weakly reduce hypertension risk. There is limited evidence suggesting a potential protective effect of moderate to high coffee consumption against MetS. However, these findings are not conclusive and warrant further investigation. Preliminary evidence indicates a potential dose-response relationship between coffee consumption and a reduced risk of NAFLD, though this is based on limited data. Consistent evidence suggests a dose-response protective effect of coffee consumption against T2D and CKD, with higher intake linked to greater risk reductions; these associations are also consistent with causal relationships. The impact of coffee on heart health remains a topic of ongoing research. While the evidence is mixed, especially for CHD, coffee consumption may be associated with a reduced risk of stroke. A U-shaped relationship with CVD outcomes is possible but not definitively established. Coffee consumption is generally associated with a reduced risk of HF, particularly with moderate intake (range 2–5 cups/day). However, higher consumption levels might increase this risk. The majority of evidence does not show a significant association between coffee consumption and AF risk, although a slight protective effect of moderate coffee intake cannot be entirely dismissed. Coffee consumption is generally associated with a lower risk of all-cause mortality, with a nonlinear U-shaped relationship and the largest risk reduction observed with moderate consumption (range 1–5 cups/day). The inverse associations between coffee consumption and adverse outcomes seem consistent across age, sex, geographical regions, and coffee subtypes, underscoring the broad applicability of these findings. Overall, these findings suggest that moderate coffee consumption (potentially filtered coffee) is generally safe and may offer protective benefits against several adverse cardiometabolic outcomes; it also has the potential to contribute to extending the healthspan and increasing longevity. Future research, particularly large-scale longitudinal observational, interventional, and MR studies and mechanistic investigations, are needed to further clarify these associations and understand the underlying biological mechanisms. This will aid in developing more targeted dietary recommendations regarding coffee consumption.

## Data Availability

This is a narrative review; no new scientific data was generated, and all data are within the paper.
